# Supplementary material for: Topology-preserving smoothing of retinotopic maps
Source: PLoS Comput Biol. 2021 Aug 2;17(8):e1009216. doi: 10.1371/journal.pcbi.1009216 (PMC8360528; doi:10.1371/journal.pcbi.1009216)
Supplement: S1 Text — (DOCX) [file pcbi.1009216.s001.docx]

# S1 Text: Notations and definitions

| Notation | Definition or Explanation |
| --- | --- |
| $R^{2}$ | The variance explained. Used to evaluate the goodness of fit. $R^{2}=100(1-\int\left( \hat{y}-y \right)^{2}dt/ \int y^{2}dt)$, where $y$ is the measurement, $\hat{y}$ is the model prediction. |
| $S=\left( F_{S},V_{S} \right)$ | Cortical surface: $V_{S}$ is the vertex list. $F_{S}$ is the triangular faces list. |
| $P=\left( X,Y,Z \right)\in\mathbb{R}^{3}$ | A point on the cortical surface |
| $\boldsymbol{u}=\left( u^{\left( 1 \right)},u^{\left( 2 \right)} \right)\in\mathbb{R}^{2}$  $u=u^{\left( 1 \right)}+iu^{\left( 2 \right)}\in\mathbb{C}$ | The parametric coordinate of the cortical surface. $\boldsymbol{u}$ and $u$ are interchangeable. When using bold $\boldsymbol{u}$, we denote the vector or 2D point $\boldsymbol{u}=\left( u^{\left( 1 \right)},u^{\left( 2 \right)} \right)$. When use $u$, we denote the complex number $u=u^{\left( 1 \right)}+iu^{\left( 2 \right)}$. |
| $\boldsymbol{v}=\left( v^{\left( 1 \right)},v^{\left( 2 \right)} \right)\in\mathbb{R}^{2}$  $v=v^{\left( 1 \right)}+iv^{\left( 2 \right)}\in\mathbb{C}$ | The visual coordinate. $v^{\left( 1 \right)}$ is eccentricity, and $v^{\left( 2 \right)}$ is polar angle. |
| $\hat{v}=\hat{v}^{\left( 1 \right)}+i\hat{v}^{\left( 2 \right)}\in\mathbb{C}$ | The smoothed visual coordinate. |
| $\sigma\in\mathbb{R}^{+}$ | Population reception field size. |
| $\Delta u_{1}u_{2}u_{3}$ or $[u_{1},u_{2},u_{3}]$ | A triangle consisted of vertices $u_{1}$, $u_{2}$, and $u_{3}$. |
| $A_{T}=\mathrm{area}\left( \Delta u_{1}u_{2}u_{3} \right)$  $A_{T}=\vert[u_{1},u_{2},u_{3}]\vert$ | The area of a triangle consisted of vertices $u_{1}$, $u_{2}$, and $u_{3}$. |
| $\mu=\rho+i\tau$ | Beltrami coefficient $\mu=\frac{\partial f/\partial\bar{z}}{\partial f/\partial z}$, where $\rho, \tau$ are real numbers. |
| $\nabla=\left( \partial/\partial u^{\left( 1 \right)},\partial/\partial u^{\left( 2 \right)} \right)$ | The gradient operator in the planar domain. |
| $\boldsymbol{\nabla}\cdot$ | Divergence $\boldsymbol{\nabla}\cdot$on vector $\boldsymbol{G}=\left( G^{\left( 1 \right)},G^{\left( 2 \right)} \right).$ In the 2D planar domain, it is calculated as $\boldsymbol{\nabla}\cdot\boldsymbol{G}=\partial G^{\left( 1 \right)}/\partial u^{\left( 1 \right)}+\partial G^{\left( 2 \right)}/\partial u^{\left( 2 \right)}$. |
| Topological/Topology-preserving | The topological relationship maintained in retinotopic mapping. |
| PSNR | Peak Signal Noise Ratio, in the unit of dB. |

**Table A.** List of symbols, notations, and brief definitions
