# Supplementary material for: Topology-preserving smoothing of retinotopic maps
Source: PLoS Comput Biol. 2021 Aug 2;17(8):e1009216. doi: 10.1371/journal.pcbi.1009216 (PMC8360528; doi:10.1371/journal.pcbi.1009216)
Supplement: S2 Text — (DOCX) [file pcbi.1009216.s002.docx]

# S2 Text: Beltrami coefficient

## Definition

The Beltrami coefficient associated with the complex-complex mapping $f\mathbb{: C}\to\mathbb{C}$ is defined as,

| $\mu=\frac{\partial f/\partial\bar{u}}{\partial f/\partial u} ,$ | (S1) |
| --- | --- |

where $u=u^{\left( 1 \right)}+iu^{\left( 2 \right)}$, $\bar{u}=u^{\left( 1 \right)}-iu^{\left( 2 \right)}$ and ${f\left( u \right)=f}^{\left( 1 \right)}+if^{\left( 2 \right)}$. Here, $u$ and $\bar{u}$ can be regarded as intermediate variables of independent variables $u^{\left( 1 \right)} \mathrm{and} u^{\left( 2 \right)}$ in **Eq.** (S1). According to the chain rule, we have the following expansion:

$$\begin{matrix} \frac{\partial f}{\partial\bar{u}}=\frac{\partial f}{\partial u^{\left( 1 \right)}}\frac{\partial u^{\left( 1 \right)}}{\partial\bar{u}}+\frac{\partial f}{\partial u^{\left( 2 \right)}}\frac{\partial u^{\left( 2 \right)}}{\partial\bar{u}}=\frac{1}{2}\left( \frac{\partial f}{\partial u^{\left( 1 \right)}}+i\frac{\partial f}{\partial u^{\left( 2 \right)}} \right) \\ \frac{\partial f}{\partial u}=\frac{\partial f}{\partial u^{\left( 1 \right)}}\frac{\partial u^{\left( 1 \right)}}{\partial u}+\frac{\partial f}{\partial u^{\left( 2 \right)}}\frac{\partial u^{\left( 2 \right)}}{\partial u}=\frac{1}{2}\left( \frac{\partial f}{\partial u^{\left( 1 \right)}}-i\frac{\partial f}{\partial u^{\left( 2 \right)}} \right). \end{matrix}$$

## Beltrami coefficient of a composition function

*Theorem* [30]*: Suppose* $g,f\mathbb{: C}\to\mathbb{C}$ *are mappings with Beltrami coefficients* $\mu_{g}$ *and* $\mu_{f}$*respectively. The composition mapping* $g\circ f$ *induces the Beltrami coefficient is given by,*

| $\mu_{g\circ f}=\frac{\mu_{f}+\left( \mu_{g}\circ f \right)\frac{\bar{f_{u}}}{f_{u}}}{1+\bar{\mu_{f}}\left( \mu_{g}\circ f \right)\frac{\bar{f_{u}}}{f_{u}}}.$ | (S2) |
| --- | --- |

where $\mu_{g}\circ f$ is the Beltrami coefficient for the mapping $g$.

## Proposition: Beltrami coefficients are invariant to conformal mapping.

*Proof*: The conformal transformation $g,$is a special case of quasi-conformal mapping, with the following equation $\frac{\partial g}{\partial\bar{z}}=\frac{\partial g}{\partial z}$ satisfied. According to **Eq.** (S2), the Beltrami coefficient of the composed Beltrami coefficient $\mu_{g\circ f}=\frac{\mu_{f}}{1}=\mu_{f}$. So, the Beltrami coefficient is invariant to conformal mapping. ∎

## Beltrami coefficient quantifies the mapping distortion

Geometrically, the Beltrami coefficient $\mu$ relates both the magnitude and angle dilation of a mapping function $f$. **Fig** A shows how an infinity small circle is mapped to an ellipse by function $f$. The shape distortion is related to the Beltrami coefficient. More specifically, the distortion of the mapping (the long axis over the short axis) is $K=\frac{1+\left| \mu\right|}{1-\left| \mu\right|}$ and the maximal dilation direction is half of the argument of $\mu$.

**Figure A.** The Beltrami coefficient quantifies the distortion in the mapping.

We introduce the following example to further explain BC. Let’s assume that $f$ is a uniformly linear map of $u$: $f=f^{\left( 1 \right)}+if^{\left( 2 \right)}=\left( u^{\left( 1 \right)}+2u^{\left( 2 \right)} \right)+i\left( 2u^{\left( 1 \right)}+u^{\left( 2 \right)} \right)$. Then the Beltrami coefficient for $f$ is: $\mu_{f}=\frac{\partial f/\partial\bar{u}}{\partial f/\partial u}=\left( \frac{\partial f}{\partial u^{\left( 1 \right)}}+i\frac{\partial f}{\partial u^{\left( 2 \right)}} \right)/\left( \frac{\partial f}{\partial u^{\left( 1 \right)}}-i\frac{\partial f}{\partial u^{\left( 2 \right)}} \right)=\frac{\left( 1+2i \right)+i\left( 2+i \right)}{\left( 1+2i \right)-i\left( 2+i \right)}$. Now we consider a unit circle $\left( u^{\left( 1 \right)} \right)^{2}+\left( u^{\left( 2 \right)} \right)^{2}=1$. Because $u^{\left( 1 \right)}=\left( 2f^{\left( 2 \right)}-f^{\left( 1 \right)} \right)/3$ and $u^{\left( 2 \right)}=\left( 2f^{\left( 1 \right)}-f^{\left( 2 \right)} \right)/3$, we have $\left( 2f^{\left( 2 \right)}-f^{\left( 1 \right)} \right)^{2}+\left( 2f^{\left( 1 \right)}-f^{\left( 2 \right)} \right)^{2}=9$ or ${5f^{\left( 1 \right)}}^{2}+{5f^{\left( 2 \right)}}^{2}-4f^{\left( 1 \right)}f^{\left( 2 \right)}=9$. This is an ellipse, from which one can compute the distortion and direction of maximal dilation. For a more general mapping, the local behavior is close to linear mapping. Namely, a tiny circle will be mapped to a tiny ellipse.
