# Supplementary material for: Topology-preserving smoothing of retinotopic maps
Source: PLoS Comput Biol. 2021 Aug 2;17(8):e1009216. doi: 10.1371/journal.pcbi.1009216 (PMC8360528; doi:10.1371/journal.pcbi.1009216)
Supplement: S3 Text — (DOCX) [file pcbi.1009216.s003.docx]

# S3 Text: Discrete operators on a 2D triangular mesh

We used several operators on a triangular mesh. Although most of them are described in Finite Element Analysis books [63], we briefly explain them here for a quick reference. Let $f$ be a complex function defined on a 2D planar triangular mesh $S$ and $v\boldsymbol{=}f\left( \boldsymbol{u} \right)$, its mapping values are usually stored on vertices in the discrete domain. We denote by$\boldsymbol{u=}\left( u^{\left( 1 \right)},u^{\left( 2 \right)} \right)$, $v\boldsymbol{=}v^{\left( 1 \right)}+iv^{\left( 2 \right)}$. For point $\boldsymbol{u}$ within the triangle $T_{i,j,k}$ consisted of vertices $\boldsymbol{u}_{\boldsymbol{i}}\boldsymbol{,}\boldsymbol{u}_{\boldsymbol{j}}\boldsymbol{,}$ and $\boldsymbol{u}_{\boldsymbol{k}}$, (i.e. $T_{i,j,k}=\left[ \boldsymbol{u}_{\boldsymbol{i}}\boldsymbol{,}\boldsymbol{u}_{\boldsymbol{j}}\boldsymbol{,}\boldsymbol{u}_{\boldsymbol{k}} \right]$), the mapping value can be modeled linearly. Mathematically,

| $f\left( \boldsymbol{u} \right)\left. \right\vert_{T_{i,j,k}}=B_{i}\left( \boldsymbol{u} \right)v_{i}+B_{j}\left( \boldsymbol{u} \right)v_{j}+B_{k}\left( \boldsymbol{u} \right)v_{k}.$ | (S1) |
| --- | --- |

where $v_{i}=f\left( \boldsymbol{u}_{\boldsymbol{i}} \right)$, $v_{j}=f\left( \boldsymbol{u}_{\boldsymbol{j}} \right)$, and $v_{k}=f\left( \boldsymbol{u}_{\boldsymbol{k}} \right)$, and the coefficients, $B_{i}$*,* $B_{j}$*,* and $B_{k}$ are called the barycentric coefficients. Numerical, $B_{i}\left( \boldsymbol{u} \right)$ (similarly for $B_{j}$ and $B_{k}$) is the area ratio between triangles $\left[ \boldsymbol{u,}\boldsymbol{u}_{\boldsymbol{j}}\boldsymbol{,}\boldsymbol{u}_{\boldsymbol{k}} \right]$ and $T_{i,j,k}$, i.e. $B_{i}\left( \boldsymbol{u} \right)=\left| \left[ \boldsymbol{u,}\boldsymbol{u}_{\boldsymbol{j}}\boldsymbol{,}\boldsymbol{u}_{\boldsymbol{k}} \right] \right|/A_{T}$ , where $A_{T}=\left| \left[ \boldsymbol{u}_{\boldsymbol{i}}\boldsymbol{,}\boldsymbol{u}_{\boldsymbol{j}}\boldsymbol{,}\boldsymbol{u}_{\boldsymbol{k}} \right] \right|$ is the area of triangle $T_{i,j,k}$.

## Discrete gradient

Now we can calculate the gradient $\nabla f$ within the triangle $T_{i,j,k}$:

| $\nabla f\left( \boldsymbol{u} \right)\left. \right\vert_{T_{i,j,k}}=\nabla B_{i}\left( \boldsymbol{u} \right)v_{i}+\nabla B_{j}\left( \boldsymbol{u} \right)v_{j}+\nabla B_{k}\left( \boldsymbol{u} \right)v_{k}$ | (S2) |
| --- | --- |

where $\nabla B_{i}\left( \boldsymbol{u} \right)=\boldsymbol{s}_{i}/2A_{T}$, $\nabla B_{j}\left( \boldsymbol{u} \right)=\boldsymbol{s}_{j}/2A_{T}$, and $\nabla B_{k}\left( \boldsymbol{u} \right)=\boldsymbol{s}_{k}/2A_{T}$. $\boldsymbol{s}_{i}=\boldsymbol{n}\times\left( \boldsymbol{u}_{j}-\boldsymbol{u}_{k} \right)$ denotes a vector where $\boldsymbol{n}$ is the triangular face norm**.** Similarly, $\boldsymbol{s}_{j}=\boldsymbol{n}\times\left( \boldsymbol{u}_{k}-\boldsymbol{u}_{i} \right)$ and $\boldsymbol{s}_{k}=\boldsymbol{n}\times\left( \boldsymbol{u}_{i}-\boldsymbol{u}_{j} \right)$. Now, the gradient $\nabla f$ can be written as, $\nabla f\left( \boldsymbol{u} \right)\left. \right|_{T_{i,j,k}}=\left( v_{i}\boldsymbol{s}_{i}+v_{j}\boldsymbol{s}_{j}+v_{k}\boldsymbol{s}_{k} \right)/2A_{T}$.

## Discrete *divergence*

Divergence on a 2D vector field $\boldsymbol{G=}\left( G^{\left( 1 \right)},G^{\left( 2 \right)} \right)$ in the Euclidean space is defined as,

| $\boldsymbol{\nabla}\cdot\boldsymbol{G}=\left( \frac{\partial}{\partial u^{\left( 1 \right)}},\frac{\partial}{\partial u^{\left( 2 \right)}} \right)\cdot\left( G^{\left( 1 \right)},G^{\left( 2 \right)} \right)^{T}=\frac{\partial G^{\left( 1 \right)}}{\partial u^{\left( 1 \right)}}+\frac{\partial G^{\left( 2 \right)}}{\partial u^{\left( 2 \right)}}.$ | (S3) |
| --- | --- |

In the discrete case, if we apply **Eq.** (S3) on **Eq.** (S2) directly, the divergence will be zero everywhere except on the edges. It is not a good approximation. As we discussed in the “Linear Beltrami Solver (LBS)” subsection of the method, the divergence can be approximated as:

| $\boldsymbol{\nabla}\cdot\boldsymbol{G}\left( u_{i} \right)=\frac{1}{\left\vert D \right\vert}\int_{\partial D} \boldsymbol{G\cdot}(\boldsymbol{n}\times d\boldsymbol{l})=\frac{1}{\left\vert D \right\vert}\sum_{\left[ u_{i}, u_{j},u_{k} \right]\in N\left( u_{i} \right)} \boldsymbol{G}_{T_{l}}\cdot\frac{\boldsymbol{s}_{\boldsymbol{i}}}{2}$*.* | (S4) |
| --- | --- |

We can interpret **Eq**. (S4) as follows: the diverge value on a vertex is the average out-flux of vector field $\boldsymbol{G}$ of its dual polygon.

## Discrete Laplacian

Let $v=f\left( \boldsymbol{u} \right),$the Laplacian operator for a scalar function $v^{\left( 1 \right)}$ in Cartesian coordinate is $\nabla^{2}v^{\left( 1 \right)}=\nabla\cdot\nabla v^{\left( 1 \right)}$ (Similar for $v^{\left( 2 \right)}$). As we have discussed discrete gradient and divergence, the Laplacian operator can be expressed:

| $\nabla^{2}v^{\left( 1 \right)}=\frac{1}{\left\vert D \right\vert}\sum_{\left[ u_{i}, u_{j},u_{k} \right]\in N\left( u_{i} \right)} {\nabla v^{\left( 1 \right)}\vert}_{\left[ u_{i}, u_{j},u_{k} \right]}\cdot\frac{\boldsymbol{s}_{\boldsymbol{i}}}{2}.$ | (S5) |
| --- | --- |

Furthermore, $\nabla^{2}v^{\left( 1 \right)}=0$ can be written in a matrix form: $L\boldsymbol{V}^{\left( 1 \right)}=0$ where $L$ is the discrete Laplacian-Beltrami operator, and $\boldsymbol{V}^{\left( 1 \right)}=\left[ {v_{1}}^{\left( 1 \right)},{v_{2}}^{\left( 1 \right)},\ldots,{v_{M}}^{\left( 1 \right)} \right]^{T}$ ($M$ is the number of vertices). In linear algebra, the element $L_{i,j}$ denotes the coefficient of $v_{j}^{\left( 1 \right)}$ when solving the $i$-th equation, namely $L_{i,1}{v_{1}}^{\left( 1 \right)}+L_{i,2}{v_{2}}^{\left( 1 \right)}+\ldots+L_{i,M}{v_{M}}^{\left( 1 \right)}=0$. Substitute **Eq.** (S2) into **Eq.** (S5), one can write:

| $L_{i,j}=\left\{ \begin{aligned} \sum_{\left[ \boldsymbol{u}_{i}\boldsymbol{,}\boldsymbol{u}_{j}\boldsymbol{,}\boldsymbol{u}_{k} \right]\boldsymbol{\in}N\left( i \right)} \frac{\boldsymbol{s}_{i}\cdot\boldsymbol{s}_{j}}{\left\vert\left[ \boldsymbol{u}_{i}\boldsymbol{,}\boldsymbol{u}_{j}\boldsymbol{,}\boldsymbol{u}_{k} \right] \right\vert}\boldsymbol{,} \text{if} i\neq j\text{ } \\ -\sum_{\boldsymbol{k\neq i}} L_{i,k}, \text{if} i=j \\ 0, otherwise, \end{aligned} \right.$ | (S6) |
| --- | --- |

where$N\left( i \right)$ is the set of triangles attached to vertex $i$. Notice we have dropped the coefficient $\frac{1}{2\left| D \right|}$ as it will not affect the results if we solve $L\boldsymbol{V}^{\left( 1 \right)}=0$. **Eq**. (S6) is interpreted as follows: (1) if vertex $j$ is adjacent to vertex $i$ or equivalently $\left[ \boldsymbol{u}_{i}\boldsymbol{,}\boldsymbol{u}_{j}\boldsymbol{,}\boldsymbol{u}_{k} \right]\text{ }$form a triangle, then ${f_{j}}^{\left( 1 \right)}$ contributes to the divergence. Since any edge is shared at most by two faces, the summation of the first condition has at most two terms.

## Discrete Generalized Laplacian

The LBS equation is $\boldsymbol{\nabla}\cdot A\nabla v^{\left( 1 \right)}=0$, where $A=\left( \begin{matrix} \alpha_{1} & \alpha_{2} \\ \alpha_{2} & \alpha_{3} \end{matrix} \right)$ is given. Follow the same steps, one can write the LBS equation in matrix form $Lv^{\left( 1 \right)}=0$ with its elements,

| $L_{i,j}=\left\{ \begin{aligned} \sum_{\left[ \boldsymbol{u}_{\boldsymbol{i}}\boldsymbol{,}\boldsymbol{u}_{\boldsymbol{j}}\boldsymbol{,}\boldsymbol{u}_{\boldsymbol{k}} \right]\boldsymbol{\in}\boldsymbol{N}\left( \boldsymbol{i} \right)} \frac{{\boldsymbol{(}A\boldsymbol{s}}_{j})\cdot\boldsymbol{s}_{i}}{\left\vert\left[ \boldsymbol{u}_{i}\boldsymbol{,}\boldsymbol{u}_{j}\boldsymbol{,}\boldsymbol{u}_{k} \right] \right\vert}\boldsymbol{,} \text{if} i\neq j\text{ } \\ -\sum_{\boldsymbol{k\neq i}} L_{i,k}, \text{if} i=j\text{ } \\ 0, otherwise. \end{aligned} \right.$ | (S7) |
| --- | --- |
