# Supplementary material for: Topology-preserving smoothing of retinotopic maps
Source: PLoS Comput Biol. 2021 Aug 2;17(8):e1009216. doi: 10.1371/journal.pcbi.1009216 (PMC8360528; doi:10.1371/journal.pcbi.1009216)
Supplement: S4 Text — (DOCX) [file pcbi.1009216.s004.docx]

# S4 Text: Angle Distortion

## Definition of Tangent Angle and Estimation Method

The tangent angle is the angle $\alpha$ spanned by the tangent of the eccentricity level set curve and the polar angle level set curve. In the synthetic data, the tangent is in 2D. For the HCP dataset, the angle is on the cortical surface, as shown in **Fig A**. Note that all the computations were carried out on the pial surface although we show the smoothed cortical surface in **Fig A**a for a clearer view of the results.


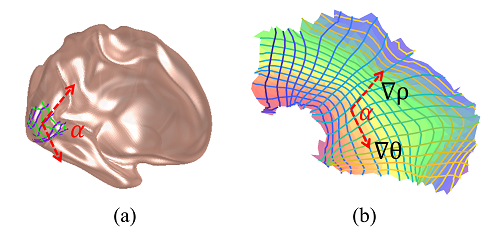


**Figure A.** Tangent Angle on the cortical surface and the parametric domain.

If we map the occipital lobe with conformal mapping, all angles are preserved. Thus, we can estimate the angle distortion in 2D, which is easier. Denote the smooth retinotopic mapping for the new parametric coordinate $u$ by $f=f^{\left( 1 \right)}\left( u \right)+if^{\left( 2 \right)}\left( u \right)$, one can estimate the angle distortion from the visual space to the cortical surface,

| $\delta_{\angle}=\left\vert\frac{\pi}{2}-\angle\left( \nabla f^{\left( 1 \right)},\nabla f^{\left( 2 \right)} \right) \right\vert=\frac{\pi}{2}-\arccos\frac{\left\langle\nabla f^{\left( 1 \right)},\nabla f^{\left( 2 \right)} \right\rangle}{\left\vert\nabla f^{\left( 1 \right)} \right\vert\left\vert\nabla f^{\left( 2 \right)} \right\vert}.$ | (S1) |
| --- | --- |

In the discrete case, since we linearly approximated the visual coordinates within each triangle, simply averaging the faces as the angle distortion on each vertex is not precise. Instead, as shown in **Fig A**, we first drew contour curves for $f^{\left( 1 \right)}$ and $f^{\left( 2 \right)}$ respectively, and then estimated the tangent vector at the intersections of the contour curves (level sets).

## Relation to Beltrami Coefficient

We further show the relationship between angle distortion $\delta_{\angle}$ and the Beltrami coefficient $\mu=\rho+i\tau$. Using the definition of $\mu$, we can eliminate $\partial f^{\left( 1 \right)}/\partial u^{\left( 2 \right)}$ and $\partial f^{\left( 2 \right)}/\partial u^{\left( 2 \right)}$ with the terms of $\partial f^{\left( 1 \right)}/\partial u^{\left( 1 \right)}$ and $\partial f^{\left( 2 \right)}/\partial u^{\left( 1 \right)}$. Eventually, the angle distortion can be written as:

$$\delta_{\angle}= \frac{\pi}{2}-\text{arccos}\left( \frac{2 \left( k^{2} \rho^{2} \tau+k^{2} \tau^{3}-k^{2} \tau+2 k \rho^{3}+4 k \rho^{2}+2 k \rho\tau^{2}+2 k \rho+4 k \tau^{2}-\rho^{2} \tau-\tau^{3}+\tau\right)}{\left( \rho^{2}+2 \rho+\tau^{2}+1 \right)^{2}+\left( k \rho^{2}+k \tau^{2}+2 \tau-k \right)^{2}} \right),$$

where $k=\frac{\partial f^{\left( 2 \right)}/\partial u^{\left( 1 \right)}}{\partial f^{\left( 1 \right)}/\partial u^{\left( 1 \right)}}$. In general, $\mu$ cannot be used to determine angle distortion $\delta_{\angle}$ without $k$. In particular, the angle distortion is $\delta_{\angle}=0$ when $\mu=0$.

## Relation to anisotropy

Angle distortion is closely related to anisotropy, but not identical. Let us assume that we have a retinotopic map that maps an infinitesimal circle in visual space to an infinitesimal ellipse on a 3D cortical surface. Since the ellipse is very small, we can assume that the 3D cortical surface is flat in this tiny region. We can cut this tiny cortical region out and place it on the disk (possibly with uniform scaling).

Anisotropy quantifies the eccentricity of the ellipse by taking the ratio of longest axis to the shortest axis, $K$. If $K=1$, a circle is mapped as circle. Although the Beltrami is used to monitor topological condition in this paper, we shall emphasize that Beltrami can also be used to compute anisotropy: $K=\frac{1+\left| \mu\right|}{1-\left| \mu\right|}$ (**Fig** B).

**Figure B.**  Illustration of anisotropy in retinotopic map and its relation to the Beltrami coefficient.

**Figure C.** Illustration of angle distortion in retinotopic map.

Angle distortion is related to the anisotropy. Only if the retinotopic map is conformal, one can use anisotropy to directly infer angle distortion.

We further show the relationship between angle distortion $\delta_{\angle}$ and the Beltrami coefficient $\mu=\rho+i\tau$. Using the definition of $\mu$, we can eliminate $\partial f^{\left( 1 \right)}/\partial u^{\left( 2 \right)}$ and $\partial f^{\left( 2 \right)}/\partial u^{\left( 2 \right)}$ with the terms of $\partial f^{\left( 1 \right)}/\partial u^{\left( 1 \right)}$ and $\partial f^{\left( 2 \right)}/\partial u^{\left( 1 \right)}$. Let and $k=\frac{\partial f^{\left( 2 \right)}/\partial u^{\left( 1 \right)}}{\partial f^{\left( 1 \right)}/\partial u^{\left( 1 \right)}}$, angle distortion can be written as: $\delta_{\angle}= |\frac{\pi}{2}-\alpha|$, where $\alpha=\text{arccos}\left( \frac{2 \left( k^{2} \rho^{2} \tau+k^{2} \tau^{3}-k^{2} \tau+2 k \rho^{3}+4 k \rho^{2}+2 k \rho\tau^{2}+2 k \rho+4 k \tau^{2}-\rho^{2} \tau-\tau^{3}+\tau\right)}{\left( \rho^{2}+2 \rho+\tau^{2}+1 \right)^{2}+\left( k \rho^{2}+k \tau^{2}+2 \tau-k \right)^{2}} \right)$ (**Fig C**).

In general, $\mu$ cannot be used to determine angle distortion $\delta_{\angle}$ without $k$. However, the anisotropy ratio can be determinate by $\mu$ directly: $K=\left( 1+\rho^{2}+\tau^{2} \right)/\left( 1-\rho^{2}-\tau^{2} \right)$. In particular, angle distortion is $\delta_{\angle}=0$ and $K=1$ when $\mu=0$ (when the retinotopic map is conformal).
