# Supplementary material for: Topology-preserving smoothing of retinotopic maps
Source: PLoS Comput Biol. 2021 Aug 2;17(8):e1009216. doi: 10.1371/journal.pcbi.1009216 (PMC8360528; doi:10.1371/journal.pcbi.1009216)
Supplement: S5 Text — (DOCX) [file pcbi.1009216.s005.docx]

# S5 Text: Supplementary data and code

## Flipped triangles in V1

We listed the number of flipped triangles of the first five observers in **Tab A**. The results for all observers can be found on the Open Science Foundation website <https://osf.io/dbgkf/>.

| Observers | Raw | Ave. | Med. | Lap. | Ours |
| --- | --- | --- | --- | --- | --- |
| S1 | 118 | 55 | 63 | 63 | **0** |
| S2 | 124 | 66 | 77 | 77 | **0** |
| S3 | 90 | 24 | 34 | 34 | **0** |
| S4 | 93 | 7 | 16 | 16 | **0** |
| S5 | 102 | 18 | 43 | 43 | **0** |

**Table A. The number of flipped triangles for the first five observers after smoothing with different methods. The total number of triangles is 548.**

## Smoothed retinotopic maps in V1/V2/V3

The smoothed retinotopic maps of the first three subject in the V1/V2/V3 complex are shown in **Fig** A. The results of all observers can be found on the OSF website <https://osf.io/dbgkf/>.


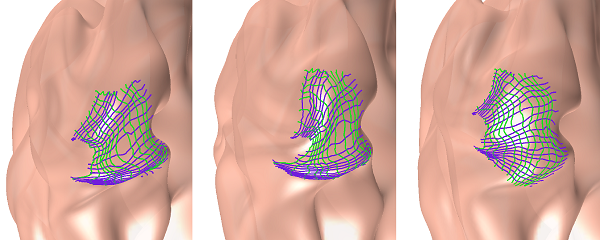


**Figure A**. The smoothed retinotopic maps of the first three observers in the V1-V2-V3 complex (left hemisphere).

## New boundaries

The visual area boundaries of the first three observers from the proposed method are shown in **Fig** B. The results of all observers can be found on the OSF website <https://osf.io/dbgkf/>.

**Figure B**. Delineate boundaries according to the smoothed retinotopic maps of the first three observers.

## Right hemisphere

Although we show the results on the left hemisphere, results on the right hemisphere are available on the OSF website <https://osf.io/dbgkf/>.

## Code

The executable program for reproducing figures, performing analyses, and a step-by-step introduction are available on the OSF website <https://osf.io/dbgkf/>.
