# Supplementary material for: Topology-preserving smoothing of retinotopic maps
Source: PLoS Comput Biol. 2021 Aug 2;17(8):e1009216. doi: 10.1371/journal.pcbi.1009216 (PMC8360528; doi:10.1371/journal.pcbi.1009216)
Supplement: Attachment — Submitted filename: ResponseLetter-Final.pdf [file pcbi.1009216.s006.pdf]

Ira A. Fulton Schools of Engineering
Arizona State University

NEW YORK UNIVERSITY

May 16, 2021

Drs. Feilim Mac Gabhann and Jason Papin
Editors-in-Chief, *PLOS Computational Biology*

Dear Drs. Robinson and Einhäuser,

Thanks for reviewing our manuscript entitled *"Topology-Preserving Smoothing of Retinotopic Maps" (PCOMPBIOL-D-20-02101)*.

We thank the reviewers for spending time to review our manuscript and offering valuable and constructive comments. We are delighted that the reviewers are generally positive about the paper. We found their comments very helpful and have revised the manuscript to address all their concerns (significant updates are in **bold**). The point-by-point responses to individual comments are appended. We believe the revised manuscript is ready for publication in PLoS Computational Biology.

Sincerely,

Yanshuai Tu, Duyan Ta, Zhong-Lin Lu, and Yalin Wang

Reviewer #1: *The paper presents a method for retinotopic maps that preserve topology. The Beltrami coefficient is used to define the topology.*

Response: Thanks for the excellent overview. The Beltrami equation is a powerful modeling tool, especially for 2D-to-2D mappings. We believe it is capable of solving many problems in noisy retinotopic mapping.

Q1.1 *Why use Beltrami coefficient? Surface Jacobian can be computed directly and that could be a more direct measure of distortion. In fact, surface Jacobian has been used by the authors in the past.*

Response: We agree that both Jacobian and Beltrami coefficients can be used to quantify the topological condition. The topological condition $||\mu||_\infty < 1$ is closely related to the determinant of Jacobian because $J = |f_z|^2 - |f_{\bar{z}}|^2 = |f_z|^2(1 - |\mu|^2)$. We prefer the Beltrami coefficient because it is more suitable for retinotopic mapping. *First*, with the Beltrami coefficient, we can define the topological constraint as a penalty term to smoothing energy (**Eq.** 7) because the Beltrami coefficient quantifies angle distortions, and retinotopic mapping is considerably angle preserving (Adams and Horton, 2003; Schwartz, 1980). In contrast, J cannot be added as a term in smoothing energy because it quantifies area changes, and retinotopic mapping is not area-preserving. Numerically, one must separately treat the constraint and adjust visual coordinates to ensure that J is positive. *Second*, as indicated by Theorem 1 in Supplementary Information (SI) 2, the Beltrami coefficient map uniquely determines a diffeomorphic mapping between two 2D surfaces, making it possible to recover/solve the mapping if we know the Beltrami coefficients. Therefore, we can divide the smoothing process into two parts and solve each part separately and efficiently. To our knowledge, there are no efficient and stable methods to utilize Jacobian J to reconstruct diffeomorphic mappings. In summary, although it may be possible to use J to quantify the topological condition, the Beltrami coefficient is more suitable for modeling retinotopic maps.

Revision: We have added a subsection in the Discussion section:
"***Beltrami coefficient vs. Surface Jacobian***
In this work, the topological condition is ensured by enforcing $||\mu||_\infty < 1$. Another geometric concept, the determinant of the Jacobian matrix J can also be adopted to enforce the topological condition. For example, it has been adopted by previous work to monitor the local visual field signs of retinotopic maps [14], which indicate the trend of visual polar angle changes (increasing or decreasing) on an iso-level eccentricity curve. Constraining the determinant of the Jacobian to be positive can also ensure the topological condition and visual field sign within a neighborhood. Mathematically, the Beltrami coefficient and the determinant of the Jacobian matrix are related via the following equation: $J = |f_z|^2 - |f_{\bar{z}}|^2 = |f_z|^2(1 - |\mu|^2)$ [59].

We prefer the Beltrami coefficient because it is more suitable for retinotopic mapping. *First*, with the Beltrami coefficient, we can define the topological constraint as a penalty term to smoothing energy (Eq. 7) because the Beltrami coefficient quantifies angle distortions and retinotopic mapping is considerably angle preserving [53,60]. In contrast, J cannot be added as a term in smoothing energy because it quantifies area changes, and retinotopic mapping is not area-preserving. Numerically, one must separately treat the constraint and adjust visual coordinates to ensure that J is postive. *Second*, as indicated by Theorem 1 in

Supplementary Information 2, the Beltrami coefficient map uniquely determines a diffeomorphic mapping between two 2D surfaces, making it possible to recover/solve the mapping if we know the Beltrami coefficients. Therefore, we can divide the smoothing process into two subproblems and solve each separately and efficiently. To our knowledge, there are no efficient and stable methods to utilize Jacobian J to reconstruct diffeomorphic mappings. In summary, although it may be possible to use J to quantify the topological condition, the Beltrami coefficient is more suitable for modeling retinotopic maps. "

Q1.2 *More description of Beltrami coefficient should be added to the paper.*

Response and Revision: Thank you for the suggestion. The following updates have been made in the revised manuscript.

(1) We've added detailed definition and properties of the Beltrami coefficient in the Method section.

"Quasiconformal mapping and Beltrami coefficient

Most surface mappings can be modeled by quasi-conformal mapping, a generalization of conformal mapping. Specifically, the local deformation of a quasiconformal map can be characterized by its associated Beltrami coefficient [39]. Mathematically, $f: \mathbb{C} \to \mathbb{C}$ is quasiconformal if it satisfies the Beltrami equation,

$$\frac{\partial f}{\partial \bar{u}} = \mu_f \frac{\partial f}{\partial u} \quad (3)$$

for some complex-valued Lebesgue measurable function μ_f satisfying $\|\mu_f\|_\infty < 1$. The complex-valued $\mu_f = \frac{\partial f}{\partial \bar{u}} / \frac{\partial f}{\partial u}$ is called the Beltrami coefficient of f, with $u = u^{(1)} + iu^{(2)}$ and $\bar{u} = u^{(1)} - iu^{(2)}$.

Intuitively speaking, if $\|\mu_f\|_\infty < 1$, the mapping preserves the orientations of all the triangles and therefore preserves the topological condition. However, if $\|\mu_f\|_\infty > 1$ the topological condition is violated because the orientations of some of the triangles are flipped. "

(2) We enriched the geometry interpretation of the Beltrami coefficient in Supplementary Information 1 by adding a specific example.

"**We introduce the following example to further explain BC. Let's assume that f is a uniformly linear map of u: $f = f^{(1)} + if^{(2)} = \left(u^{(1)} + 2u^{(2)}\right) + i\left(2u^{(1)} + u^{(2)}\right)$. Then the Beltrami coefficient for f is: $\mu_f = \frac{\partial f/\partial \bar{u}}{\partial f/\partial u} = \left(\frac{\partial f}{\partial u^{(1)}} + i\frac{\partial f}{\partial u^{(2)}}\right) / \left(\frac{\partial f}{\partial u^{(1)}} - i\frac{\partial f}{\partial u^{(2)}}\right) = \frac{(1+2i)+i(2+i)}{(1+2i)-i(2+i)}$. Now we consider a unit circle $\left(u^{(1)}\right)^2 + \left(u^{(2)}\right)^2 = 1$. Because $u^{(1)} = \left(2f^{(2)} - f^{(1)}\right)/3$ and $u^{(2)} = \left(2f^{(1)} - f^{(2)}\right)/3$, we have $\left(2f^{(2)} - f^{(1)}\right)^2 + \left(2f^{(1)} - f^{(2)}\right)^2 = 9$ or $5{f^{(1)}}^2 + 5{f^{(2)}}^2 - 4f^{(1)}f^{(2)} = 9$. This is an ellipse, from which one can compute the distortion and direction of maximal dilation. For a more general mapping, the local behavior is close to linear mapping. Namely, a tiny circle will be mapped to a tiny ellipse.**"

Q1.3 *How does the Beltrami coefficient relate to the topology and distortion should be described in more detail possibly with some illustrations.*

Response and Revision: Thank you for the suggestion. It is a key concept. The following updates have been made in the revised manuscript.

(1) We've added a description of the relationship between topological condition and Beltrami coefficient in the Method section:

"Intuitively speaking, if $\|\mu_f\|_\infty < 1$, the map preserves the orientations of all triangles and therefore preserves the topological condition. However, if $\|\mu_f\|_\infty > 1$, the topological condition is violated because the orientations of some triangles are flipped."

(2) We've updated **Fig. 2** and added more descriptions to describe how μ_f's value relates to the topological condition. Namely, the value and its mapping target are explained.

"**In Fig. 2b, we illustrate three mapping results for P_i: Case (1) if f_i coincides with the green dot such that triangles $\Delta P_i P_j P_k$ and $\Delta f_i f_j f_k$ are similar ($\Delta P_i P_j P_k \sim \Delta f_i f_j f_k$), the magnitude of the Beltrami coefficient associated with triangle $\Delta P_i P_j P_k$ is zero ($|\mu| = 0$). This is the case of conformal mapping. Case (2) if f_i is above the $f^{(1)}$ axis ($f_i^{(2)} > 0$), shown as the yellow dot, the magnitude of the Beltrami coefficient associated with triangle $\Delta P_i P_j P_k$ is greater than zero but less than one ($0 < |\mu| < 1$). This is the case of quasiconformal mapping with respect to edge $\overline{f_j f_k}$. And Case (3) if f_i is below the $f^{(1)}$ axis, shown as the red dot, the magnitude of the Beltrami coefficient associated with triangle $\Delta P_i P_j P_k$ is greater than one ($|\mu| > 1$) and the mapping is no longer topological. In summary, the complex-valued Beltrami coefficient encodes relative mapping information for each triangle and can be used to quantify the topological condition [32,33]**."

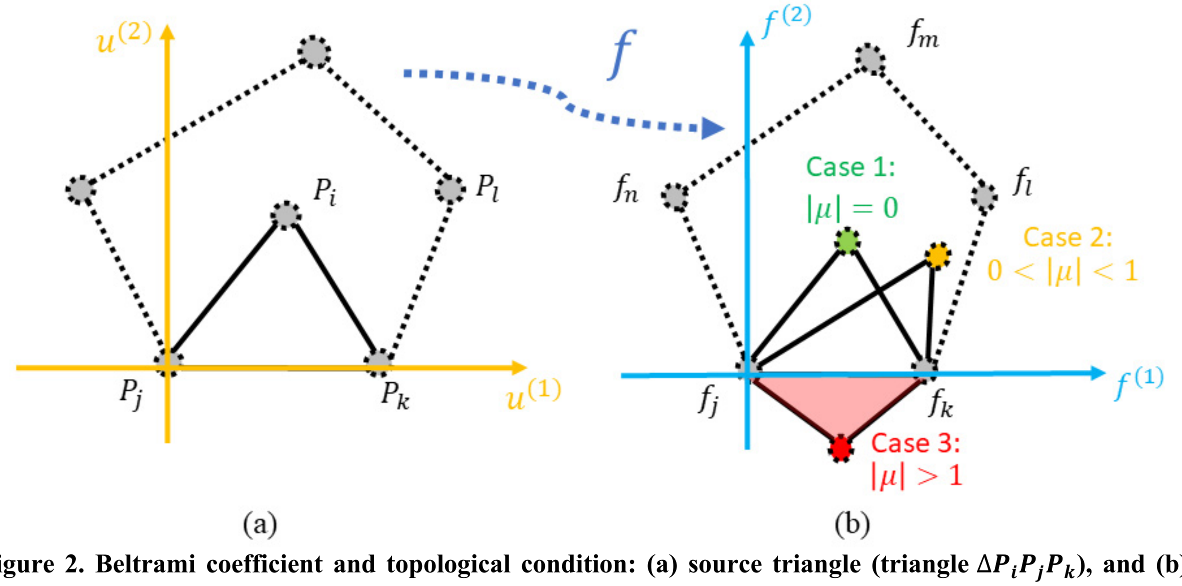

Figure 2. Beltrami coefficient and topological condition: (a) source triangle (triangle $\Delta P_i P_j P_k$), and (b) mapping results of the source triangle, triangle $\Delta f_i f_j f_k$. Three different mapping cases f_i result in different Beltrami coefficients.

Q1.4 *How does the smoothing in fMRI data affect the Beltrami coefficient?*

Response: Here, we assume that by "fMRI data smoothing" you meant smoothing of visual coordinates in this study.

The "raw" retinotopic map is about 80% topology preserving (See **Table S2**: about 80% of triangles have $|\mu| < 1$ before smoothing). Because the Beltrami coefficient quantifies local deformations in retinotopic mapping, the smoothing reduces the number of very large BCs and reduces the norms of the Beltrami coefficients.

Revision: We added the following description to the manuscript in the *Solving the first subproblem: Laplacian smoothing* subsection:

"Since the Beltrami coefficients quantify local deformations, Laplacian smoothing on visual coordinates will smooth the Beltrami coefficients. If most triangles are correctly orientated, the smoothing will also reduce the number of flipped triangles."

Q1.5 *I think the notation of first fundamental form in eq 3 might not be necessary. If the authors want to keep it, more description of them should be added.*

Response and Revision: Thank you for the suggestion. We've removed the description of the first fundamental form in Eq 3. To accommodate the change, we introduced the parametrization before the computation of Beltrami coefficients. We introduced the following changes to define the Beltrami coefficient:

First, we've moved surface parametrization before the Beltrami coefficient:

"Let f_r: $v_i \mapsto P_i$ be the retinotopic map between the retina and V1 cortical surface. To use the tool of the Beltrami coefficient, we need to flatten …."

Second, we've re-described the smoothing model with the simplified Beltrami coefficient, (Beltrami coefficient for f, where f is the mapping between the parametric disk and visual disk).

"**With the conformal parametrization in the last section, we use the Beltrami coefficient μ_f to model topological smoothing by finding new visual coordinates $\hat{v} \equiv \hat{f}$ such that,**

$$\hat{v} = \hat{f} = \arg\min_{\hat{v}} \int w\left|\hat{f} - v\right|^2 + s\left|\nabla\hat{f}\right|^2 \mathrm{ds}, \ \mathrm{s.t.} \left\|\mu_{\hat{f}}\right\|_\infty < 1. \quad \mathbf{(4)}$$

where w is the weight function, $\nabla = \left(\partial/\partial u^{(1)}, \partial/\partial u^{(2)}\right)$ is the gradient operator, s is a positive scalar emphasizing the smoothness and $\mu_{\hat{f}}$ is the Beltrami coefficient of $\hat{f}$ written as (see *SI 2*)… "

Q1.6 *Overall, this is a very interesting work. Addressing the above issues would improve the paper.*

Response: Thanks again for the encouragement.

Reviewer #2: *The manuscript by Tu, Ta, Lu & Wang introduces and develops a new method for analysing retinotopic maps. Essentially, the method enforces a topological mapping from retina/visual field space to cortical space, by using the Beltrami coefficient. Turning a measurement of many independent and noisy estimates into a consistent mapping bears many advantages and this has been previously recognized by Doughtery et al 2003 and Schira et al. 2007, using a technically different approach of morphing a map template on retinotopic data. The procedure suggested here has many advantages and some disadvantages over previous methods. The approach suggested seems very elegant and promising, the implementation points to great skills, and the method could indeed significantly improve our analysis of retinotopic maps. However, I am struggling with the current manuscript both in clarity but also structure. I suggest the authors make the purpose clear and structure the manuscript accordingly.*

Response: Thank you for the nice overview. Previous work in the area (Doughtery et al 2003, Schira et al. 2007, etc.) was efficient and robust and has greatly inspired our work. In the revised manuscript, we have made a lot of efforts to improve the structure and clarity of the manuscript.

Q2.1 *Importantly, measures and analysis such as CMF or quality assessment need to be significantly improved or removed from the manuscript. At present they seem like an afterthought and that does neither these assessments nor the method justice.*

Response and Revision: Based on our understanding of **Q2.12**, we think you meant the CMF and angle distortion subsections. Those sections were in the original manuscript to demonstrate that our method is robust and can be applied to address some important questions in vision research. We agree with you that they may distract the readers from the methodological focus the work. We've removed them in the revised manuscript.

Q2.2 *The authors should do a better job explaining their core assumption that the mapping between retina/visual field and cortex is topological. I feel Fig 1 is ill suited and instead confusing in this context. Upon reading Fig 1 (for example F1b and F1c) I was trying to understand what topological concept the six nodes and eight edges in F1c violate, for example they could form a handle or a hole in the surface. However, that little set of triangles is a perfectly fine surface if rotated. F1d is really not adding anything either. Essentially the concept is that a sequence of locations on the retina, such as a,b,c are represented in this order on cortex and not for example as a,c,b. This is obviously hard to illustrate in a figure without retinal space. The same applies to F2, where I am not certain what it explains.*

Response: Thank you for the suggestions and feedbacks.

The major motivation for us to develop tological smoothing is to enforce one of the fundamental properties of retinotopic maps observed in neurophysiology: nearby neurons have receptive fields at nearby locations on the retina [18]. We call this property the *topology-preserving condition* (or *topological condition* for short). The term "topology-preserving" was borrowed from some existing work [19]: *"Topology Preservation: Preservation of the neighbourhood relation of the observations in the output space. It means that the observations which are neighbours in the input space should be projected in neighbour nodes."* The topological condition we discussed here does not mean handles, holes, or boundaries of surfaces. It refers to the preservation of neighborhood relationships in geometric mapping.

In the revised manuscript, we've tried to explain the term with an example in **Fig. 1**. **Fig. 1a** shows a polygon structure in the source domain (V1 cortical surface). If the retinotopic map is topological, it should preserve the relative neighborhood positions in the target domain (retina space). Namely, a topological map may distort the size of the polygon or the relative positions of its vertices but will keep the neighborhood structure (**Fig. 1b**). However, if the map is not topological, it breaks the neighborhood structure (**Fig. 1c**). In **Fig. 1b**, we further show that, for a non-topological map, the orientation of at least one triangle is flipped (more specifically, the orientations are different in the source and target domains). We use red color to highlight the *flipped triangles* in **Fig. 1d**. A topology-perserving retinotopic mapping from the visual cortex to the retina field should have no flipped triangle. However, 20% (see **Table S2**) of triangles in a typical empirical retinotopic map are flipped (**Fig. 1d**).

In **Fig. 2** of the revised manuscript, we added three colored points to illustrate three cases of geometric mapping. Cases 1 and 2 are topological, while case 3 is non-topological.

Revision: In the second paragraph of the revised Introduction, we defined the concept of topological condition and explained **Fig. 1** in detail.

"More specifically, neighboring points on the cortical surface should have neighboring retinal visual coordinates [18]. We adopted the term *topology preserving (or topological)* to refer to the preservation of neighborhood relationships of the observations in the output space [19]. In this work, the topological condition or topology-preserving are not related to the surface genus, handles or holes. The concept of topological condition is illustrated in Fig. 1 for retinotopic mapping. Figs. 1ab show an example of topological mapping that preserves the structure of the source polygon (on the cortical surface) in the target (retina) domain (Fig. 1b). Figs. 1ac show an example of non-topological mapping that breaks the source polygon structure after mapping: f_i is out of the polygon edge $\overline{f_j f_k}$, resulting in what is called a *flipped triangle* $\Delta f_i f_j f_k$ (the orientations of the triangle are different in the source and target domains)."

We've updated **Fig. 1** caption to better explain the pictures. We've also updated **Fig. 2** by adding more notations.

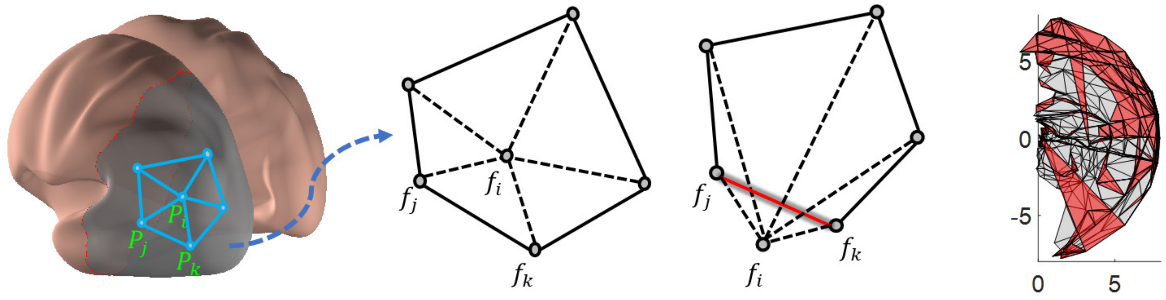

(a) (b) (c) (d)

Figure 1. Illustration of topological and non-topological **maps**. (a) A polygon patch on an inflated cortical surface (source domain). (b) A topological mapping of (a) **in the target domain (retina space)**. It preserves the structure of the source polygon. (c) A non-topological mapping of (a). It breaks the structure of the source polygon: f_i is moved out of the edge $\overline{f_j f_k}$, resulting in what is called a *flipped triangle* $\Delta f_i f_j f_k$. (d) A typical retinotopic map of V1. Flipped triangles are labeled with red color.

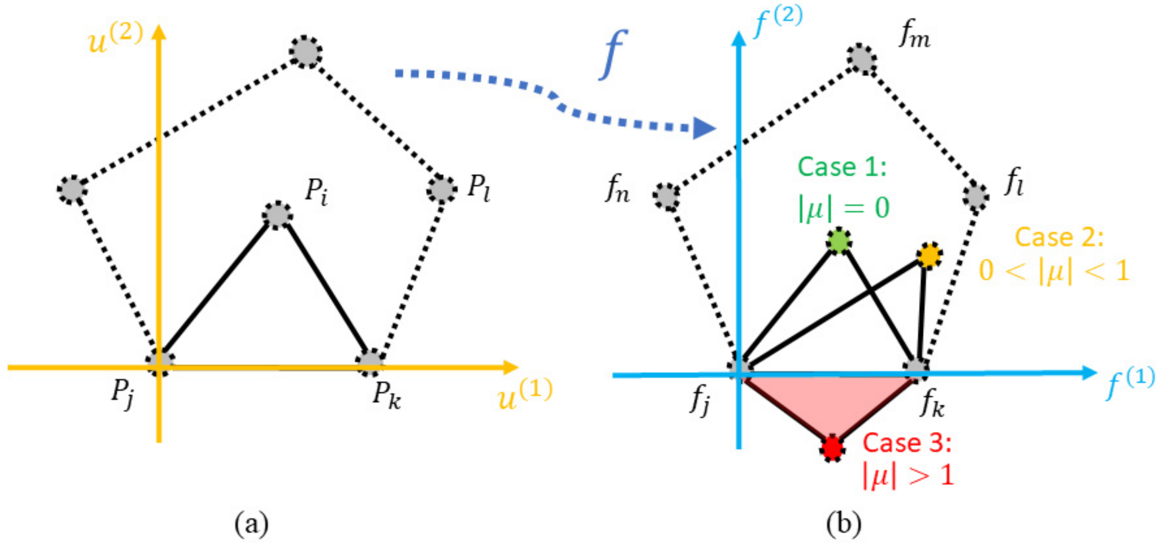

Figure 2. Beltrami coefficient and topological condition: **(a) source triangle (triangle $\Delta P_i P_j P_k$), (b) three target triangles $\Delta f_i f_j f_k$ from three different types of mappings, with different Beltrami coefficients.**

Q2.3 *The project starts with the proposal that no assumptions are needed other than enforcing the "topological condition", but unfortunately this is not exactly correct. In order to map across several visual areas, the accuracy of boundary labels is a major concern. An ill placed boundary can significantly impact the smoothing process, by introducing apparent breaches in topology that do not exist in the data. The section "Robustness with inaccurate interior boundaries" L664f tries to address this, but the section is entirely insufficient and has a number of flawed assumptions. I was also struggling with comprehension of this section.*

Response: Thank you for the feedback. We believe it is one of the key questions that deserves more clarification. We distinguish two different boundaries in this paper. While interior boundaries are those between different visual areas (V1 and V2, V2 and V3, etc), exterior boundaries separate the visual areas under consideration from the rest of the brain, such as V3 boundaries and the boundaries defined along the out-most eccentricity curves. In this answer, we focus on the interior boundaries; we discuss exterior boundaries in the response to **Q3.7** below.

The delineation of interior boundaries of visual areas is a very important issue. In our work, the initial interior boundaries of visual areas are obtained with multimodal surface registration developed in the HCP [56,57]. We cannot assume they are accurate. Our proposed algorithm is designed to be robust to inaccurate boundaries.

We employ a concept, the *extended polar angle,* to encode both polar angle and visual area labels, which can be transformed back to the original polar angle and visual area labels. Therefore, with accurate extended polar angles, one can draw accurate interior boundaries between different visual areas (V1 and V2, V2 and V3, etc). An ill placed visual boundary would significantly impact the extended polar angle (generating a "spike") and violate the topological condition. By checking topological violations, one can detect the spikes caused by incorrect boundary placement. On the other hand, "fixing" the incorrect extended polar angles would improve interior boundaries between different visual areas.

To fix the incorrect extended polar angles, we used two principles: the goodness of fit and topological condition (that is, the mapping from cortex to the extended visual coordinates is

topology-preserving). Specifically, the corrected extended visual coordinates should be close to the original pRF coordinates and have no violoation of the topological condition. With conventional smoothing, e.g., Laplacian smoothing, one cannot ensure the topological condition. In our proposed framework, we add a topology-projection module (subproblem 2) to Laplacian smoothing (subproblem 1). If the initial boundary is incorrect, it would violate the topological condition of the 2D retinotopic map from the parametrization domain to the extended visual coordinate domain. The topology-projection fixes such topological-violations by adjusting the visual coordinates. Laplacian smoothing will then try to smooth the results. With iterations, the incorrect boundary is fixed with smoothed interpretations of nearby values. The result balances between topology-projection and Laplacian smoothing.

Revision: In "Removing phase reversal" subsection of Methods, we've added the following discussion.

"Because polar angles and visual area labels have a one-to-one correspondence with the extended polar angles, one can recover the original polar angles and visual area labels from the extended polar angles. With accurate extended polar angles, one can draw accurate interior boundaries between different visual areas (V1 and V2, V2 and V3, etc)."

We've refined the discussion of **Fig. 14** in "Robust to inaccurate interior boundaries" subsection of Discussion.

"We further verified that the robustness was not due to Laplacian smoothing. In **Fig.** 14c, we plot the raw data and topologically smoothed results of the white curve. **The white curve is formed by points with eccentricity $v^{(1)} = 0.3°$ with tolerance $0.05°$. We now consider the data near the white curve. If the eccentricity measurement is accurate, the 2D topological condition can be considered as a monotonicity requirement.** In **Fig.** 14c, the blue dots are the raw **extended polar angle** data, and the green curve is the topological smoothing result. Even though there were outliers (the red circled points, **mainly due to the noisy eccentricity data**), the **main portion of the trend** was monotonic. In **Fig**. **14d**, we plot the results with **only Laplacian smoothing** without topology constraints as the green curve, which is crooked **in order to fit the data** but not monotonic (topological). This means Laplacian smoothing **can smooth but cannot fix topological violations**. In contrast, our topological smoother ensures topological condition, which essentially makes the mapping "monotonic". It is the topological constraint rather than Laplacian smoothing that makes the smoothing more robust to inaccurate interior boundaries.

The new topology-projection module in the proposed method makes it robust to inaccurate interior boundaries. The smoothed extended polar angles from topological smoothing can be used to infer visual area labels."

Q2.4 *Specifically, the authors imply that their method is robust against such errors, but it is entirely unclear what they mean by this. Will mislabelling a) simply be "smoothed over without larger consequences" or if b) the method somehow corrects these mistakes and instead finds the most correct boundary. I suspect it is a). If it is indeed b) the mechanism by which this is done needs to be clearly specified. In fact it is unclear how area labels are created in the very first place.*

Response: The answer is (b). This question is highly related to **Q2.2**. Mislabeling makes the extended polar angle very different from its neighbors' (because it adds or subtracts 1π). The

topology projection module can detect and fix those "outliers", and produce continuous extended polar angles. The new extended polar angles encode the improved boundary.

In addition, we have benefited a lot from the multimodal registration framework developed by the HCP group, which generated high quality initial interior boundaries. We only needed to apply our method once.

Revision: We've added the source of the initial area labels:

"We took the initial visual area labels from the multimodal surface matching (MSM) results of the HCP group [56,57]"

Q2.5 *Assuming it is a) error will inevitably distort/smooth surrounding mapping, confounding attempts to quantify local CMF. It would be important to quantify these distortions, for example by systematically moving internal boundaries such as the V1/V2 border by medium to small amounts (15mm?) and investigate and quantify the effects.*

Response and Revision: Thank you for the suggestion. We took approach (b). We fixed incorrect interior boundaries. We appreciate the suggestion on boundary perturbation. We've added an experiment to evaluate the influence of different levels of boundary misplacement and written it up in the "Robustness with inaccurate interior boundaries" subsection of Discussion.

"We computed visual coordinate changes following an alteration of the V1/V2 boundary. If the visual coordinates did not change too much when the boundary was altered, the results were robust to interior boundaries. We started from the first subject's data (Fig. 15a). We expanded V2v by moving points in V1v into V2v if they were adjacent to V2v. The expansion was repeated to simulate different boundary misplacements (three rounds in Fig. 15b and five rounds in Fig. 15c). Then we applied our smoothing method and compared the results with no perturbation. The boxplot in Fig. 15d shows that the visual coordinate differences were relatively small (the average is less than 0.5°) with V2v boundary expansion. The results suggest that the proposed method is robust to interior boundary placement.

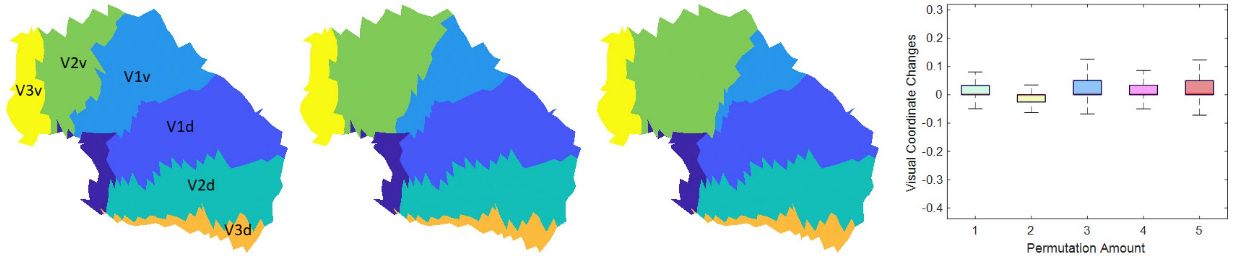

(a) (b) (c) (d)

Figure 15. (a, b, c) Perturbations of the V1/V2 boundary, where (b) was obtained after three rounds of expansion and (c) was obtained after five rounds of expansion. (d) Visual coordinate changes with different degrees of perturbations."

Q2.6 *Would it be possible to conceive a method that optimises the boundary placements? Maybe even use the Beltrami coefficient to find the optimal boundaries?*

Response: Thanks for the suggestion. The answer is yes. In the method presented here, we can adjust the interior boundaries to fix incorrect placements (see answers to **Q2.2**, **Q2.3** and **Q2.4**).

Another method is by manipulating the Beltrami coefficients of the registration function between a subject and a template (Note that the Beltrami coefficient in this sentence is for the mapping from the subject's parametric disk to the template's parametric disk, different from the Beltrami coefficient in the rest of this paper). The registration method has various advantages [8]. However, the challenge is how to generate the right template. We reported our preliminary retinotopic map registration results in [62]. Our ongoing work integrated registration and topological smoothing and achieved promising results. We will report our work in our forthcoming manuscript [61].

Revision: We've added a paragraph in Discussion.
"Relation to registration
The Beltrami coefficient (BC) encodes 2D-to-2D mapping up to a normalization [59]. Manipulating the Beltrami coefficients of the registration function between a subject and a template can also achieve diffeomorphic registration and generate topological results [62]. The benefits of this approach was discussed in [8]. However, one main issue is that the template itself might be biased. One potentially promising solution is to combine registration and smoothing: while registration provides good boundaries, topological smoothing provides good interior smoothing [61]. "

Q2.7 *It remains unclear throughout the entire manuscript what Software (Language) the authors used for implementation. I had to go to the OSF site of the project to find matlab code. Searching the document matlab is cited as reference no 51, but [51] is actually not cited in the text.*

Response and Revision: Reference [51] (in the initial version) was cited in the supplementary material. We've added the following sentence in the Algorithm subsection:
"The algorithm is implemented in MATLAB 2019b [52]."

Q2.8 *The authors apply their method to datasets of 171 participants, but for what purpose? Only the results in **Fig. 13** seem to rely on 171 datasets. It is a curious result but it breaks the consistency of the manuscript that otherwise seems to be a pure method paper.*

Response and Revision: The main purpose of presenting results on many subjects was to demonstrate the robustness of our software tool. A practical question is whether our newly developed method is capable of processing real retinotopic mapping data. That we have successfully processed the retinotopic map data from all 181 HCP observers suggests that the metod is robust.

In Discussion, we reported our results by

"Experiments with both simulated and real retinotopy data demonstrated that the proposed method could generate topological and smooth retinotopic maps."

Q2.9 *The authors describe their technique as topological smoother. To me the two things topological correctness and smoothness are not necessary the same. The name seems to suggest the method smoothes until the topological condition is met. Does that mean datasets with large inconsistencies end up being more smoothed?*

Response: It is a good point. We agree that topological correctness does not mean smoothness, nor does smoothness mean topological correctness. In fact, the topological condition can be

ensured without smoothing. Empirically, no one expects retinotopic maps are topological but extremely irregular and rough. Our method makes them both topological and smooth.

Smoothing does to some degree fix the topological condition. Although we do not know the exact relationship between smoothing visual coordinates and the topological condition (monitored by Beltrami coefficient), the Beltrami coefficient quantifies the local deformations based on its definition. Therefore, smoothing can reduce the number of outliers in Beltrami coefficients. Because 80% of the triangles in the HCP dataset are orientation preserving (See **Table S2**), smoothing the visual coordinates may reduce the norm of the Beltrami coefficients on average and improve the topological condition. If the data were worse (e.g., ~50% triangles' are flipped), smoothing may introduce more non-topological triangles and make the problem worse.

The topological condition cannot be ensured by iteratively smoothing. As shown in **Fig. 14d**, repeated Laplacian smoothing did not generate fully topological results. The topological condition is mainly fulfilled by the topological projection. However, after fixing the topological condition, a major concern is the results could be too irregular or deviate too much from the original pRF solutions. The proposed *Topological smoother* can produce results that are both topological and smooth. Roughly speaking, if the fMRI signal to noise ratio is very high (say SNR >20), the pRF solutions would be smooth and topological. For example, the pRF solution of averaged fMRI signals (https://osf.io/bw9ec/wiki/home/) has very fewer flipping triangles in V1. This is why the energy function in the topological smoother has a term that penalizes non-smoothness and therefore may improve the fit to the data and improve topological correctness. We believe the approach can balance topological correctness and smoothness.

Revision: We've added a subsection to discuss the relationship between smoothing and topological in Discussion:

"Smoothness and topological projection

The proposed topological smoother produces topological and smooth retinotopic maps. Although these two conditions are related, the topological condition does not ensure smoothness, nor does smoothness ensure the topological condition. In fact, the topological condition can be ensured without smoothing. Empirically, no one expects retinotopic maps to be topological but extremely irregular and rough.

Smoothing does to some degree improve the topological condition. Although we do not know the exact relationship between visual coordinate smoothing and the topological condition (monitored by Beltrami coefficient), the Beltrami coefficient quantifies the local deformations based on its definition. Smoothing can reduce the outliers in Beltrami coefficients. Because 80% of the triangles in the HCP dataset are orientation preserving (See Table S2), smoothing the visual coordinates may reduce the norm of the Beltrami coefficients on average and improve the topological condition. If the data have more flipped triangles (e.g., ~50% triangles' are flipped), smoothing may introduce more non-topological triangles and make the problem worse.

The topological condition cannot be ensured by reiterative smoothing. As shown in Fig. 15d, repeated Laplacian smoothing did not generate fully topological results. The topological condition is mainly fulfilled by topological projection. However, after fixing the topological condition, a major concern is the results could be too irregular or deviate too much from the original pRF solutions. The proposed Topological smoother can produce results that are both topological and smooth. Roughly speaking, if the fMRI signal to noise

ratio is very high (say SNR >20), the pRF solutions would be smooth and topological. For example, the pRF solution of the averaged fMRI signals from many subjects (https://osf.io/bw9ec/wiki/home/) has very few flipping triangles in V1. This is why the energy function in the topological smoother has a term that penalizes non-smoothness and therefore may improve the fit to the data and improve the topological condition. We believe the approach can balance the topological condition and smoothness."

Q2.10 *I was majorly struggling (failing) to follow large sections of the manuscript, in particular pages 11 to 16. I felt the authors are using a large number of symbols often inconstant and the purpose is not clear. I understand that it is hard to structure a logical problem that arose piece by piece when implementing a new method, but I feel the authors need to significantly improve this. I am unclear what the first subproblem exactly is, and what EXACTLY is smoothed. I understand what Laplacian smoothing is, but I am unclear what the mesh is that is smoothed.*

Response and Revision: Thank you for the comment. In the revised manuscript, we have updated notations, re-structured the method description and rewritten some key subsections.

First, let's briefly summarize the key ideas of our method. The goal of our work is to compute new visual coordinates to make retinotopic maps both smooth and topological by solving the following problem,

$$\hat{\boldsymbol{v}} = \hat{f} = \arg\min_{\hat{\boldsymbol{v}}} \int w\left|\hat{f} - \boldsymbol{v}\right|^2 + s\left|\nabla\hat{f}\right|^2 \mathrm{d}s,\ s.t. \left\|\mu_{\hat{f}}\right\|_\infty < 1.$$

To solve the problem efficiently, we divide the problem into two subproblems, Laplacian smoothing and topological projection. The two subproblem are related by the visual coordinates. Laplacian smoothing is a general signal smoothing method. Here, the visual coordinates are the signals to be smoothed, and Laplacian smoothing of meshes is a special case of Laplacian smoothing of signals. The visual coordinate meshes consist of $(F, \boldsymbol{v})$, where F is the triangular face list (same as the cortical triangular face list of ROI), $\boldsymbol{v}$ is the extended visual coordinates. The second subproblem is to fix the topology by computing the Beltrami coefficients and performing topological projection to enforce topological condition. All these concepts must be implemented specifically for discrete meshes. Therefore, details are introduced to explain how to discretize them with triangular meshes.

We've carefully checked the notations and updated them as follows:

(1) For simplicity, the mapping from the parametric disk to the visual field, f_c, has been changed to f, Accordingly, we changed the symbol of the retinotopic map in **Fig. 4**, Eqs. 4, 5, 10, 11,14, and the text.
(2) We've tried to use the minimum number of symbols. $\boldsymbol{u} = \left(u^{(1)}, u^{(2)}\right) \in \mathbb{R}^2$ and its complex form $u = u^{(1)} + iu^{(2)} \in \mathbb{C}$ are used to represent the coordinates on the parametric 2D disk. $\boldsymbol{v} = \left(v^{(1)}, v^{(2)}\right) \in \mathbb{R}^2$ and its complex form $v = v^{(1)} + iv^{(2)} \in \mathbb{C}$ are used to denote the corresponding visual coordinates. Similarly, $\hat{v} = \hat{v}^{(1)} + i\hat{v}^{(2)} \in \mathbb{C}$ is used to denote the smoothed visual coordinates.
(3) We've checked the consistency of hat symbols, and changed $\boldsymbol{f}$ to $\hat{\boldsymbol{f}}$ in Alg. 1; We've also check the consistency of the infinite norm:

"**Compute Beltrami coefficient $\boldsymbol{\mu}$ for the mapping $\hat{\boldsymbol{f}}: \boldsymbol{u} \rightarrow \hat{\boldsymbol{v}}$ using Eq. (5).**"

"The second subproblem is to make f topological. It means that the associated Beltrami

coefficients must satisfy the condition $\|\mu_f\|_\infty < \mathbf{1}$ on the entire cortical surface"

(4) We've updated the quasiconformal mapping and Beltrami coefficient definitions and made them more accessible.

"Quasiconformal mapping and Beltrami coefficient

Most surface mappings can be modeled by quasi-conformal mapping, a generalization of conformal mapping. Specifically, the local deformation of a quasiconformal map can be characterized by its associated Beltrami coefficient [39]. Mathematically, $f: \mathbb{C} \to \mathbb{C}$ is quasiconformal if it satisfies the Beltrami equation,

$$\frac{\partial f}{\partial \bar{u}} = \mu_f \frac{\partial f}{\partial u} \quad (3)$$

for some complex-valued Lebesgue measurable function μ_f satisfying $\|\mu_f\|_\infty < 1$. The complex-valued $\mu_f = \frac{\partial f}{\partial \bar{u}} / \frac{\partial f}{\partial u}$ is called the Beltrami coefficient of f, with $u = u^{(1)} + iu^{(2)}$ and $\bar{u} = u^{(1)} - iu^{(2)}$.

Intuitively speaking, if $\|\mu_f\|_\infty < 1$, the map preserves the orientations of all triangles and therefore preserves the topological condition. However, if $\|\mu_f\|_\infty > 1$ the topological condition is violated because the orientations of some triangles are flipped. "

(5) We used "Surface flattening" subsection to introduce our research setting.

"In this work, we focus on the 2D retinotopic mapping $f = {f_r}^{-1} \circ c^{-1}$ instead of the 3D retinotopic mapping f_r."

(6) When we introduce the mathematical model, we've tried to make them more accessible by providing some motivation and background description.

(7) We changed the order of "Model solver" and "Model discretization" subsections and rewrote these two subsections as follows.

"<u>Model solver</u>

To solve Eq. (4) efficiently, we divide the problem into two subproblems, namely smoothing and topological projection. Specifically, during smoothing, we ignore the topological condition and find a smoothed map by,

$$\tilde{v} = \arg\min_{\tilde{v}} \int w|\tilde{v} - v|^2 + s|\nabla\tilde{v}|^2 \mathrm{ds} \quad (6)$$

which can be solved by Laplacian smoothing [44,45] efficiently. For the second subproblem, we ignore the smoothness term and try to make the results topological.

$$\hat{v} = \arg\min_{\hat{v}} \int w|\hat{v} - \tilde{v}|^2 \mathrm{ds}, \mathrm{s.t.} \|\mu_{\hat{v}}\|_\infty < 1 \quad (7)$$

Since we require the results to be both topological and smooth, we solve the subproblems iteratively. Namely, the output of topological projection is fed to smoothing, and so on so forth.

The key challenge is to solve Eq. (7) efficiently. Because the topological condition is formulated with the Beltrami coefficient ($\|\mu_{\hat{v}}\|_\infty < 1$), we need tools to compute the Beltrami coefficient and recover the function after processing the Beltrami. In the following, we describe the Beltrami coefficient computation when a function is given and the function recovery when the Beltrami coefficients are given in the discrete setting. Then we elaborate on the details about solving each subproblem.

<u>Model discretization</u>

We now convert the problem to the discrete domain because a cortical surface, S, is typically described by vertices V_S and triangular faces F_S: $S = (F_S, V_S)$. The visual field is also discretized. Retinotopic mapping can be reformulated as finding the corresponding visual coordinate v_i in the visual field for every vertex $P_i \in V_S$. We denote W_i as $w(v_i)$ to emphasize/deemphasize voxels with high and low quality of pRF fits.

$$E = \sum_i W_i \left|v_i - \hat{f}_i\right|^2 + s\left|\nabla \hat{f}_i\right|^2 + \lambda|\mu_i|^2, \text{s.t.} \left|\mu_{\hat{f}_i}\right| < 1. \quad \mathbf{(8)}$$

$\hat{f}_i = \hat{f}(u_i)$ is the coordinate to be solved, $\mu_i = \mu_{\hat{f}}(u_i)$ is the Beltrami coefficient at point u_i.”

(8) For the first subproblem, we’ve added the following description to explain the connection between smoothing and topological condition.

“Since the Beltrami coefficients quantify local deformations, Laplacian smoothing on visual coordinates will smooth the Beltrami coefficients. If most triangles are correctly orientated, the smoothing will also reduce the number of flipped triangles. ”

(9) We’ve improved some sentences.

“The Beltrami of $\hat{f}$ can be used to quantify the Beltrami of ${f_r}^{-1}$ because the Beltrami coefficient does not change with the conformal mapping, that is, $\mu_f = \mu_{{f_r}^{-1}}$ if c is a conformal mapping (see *SI 2* for mathematical details).”

We hope our updates have clarified matters.

Q2.11 I *am unclear how the second subproblem is solved. Do I understand correctly that nodes with negative coefficients are found, then their coefficient is changed by “shrinking” whatever that means? Shrinking sounds like an iterative process. L337 refers to Figure 2d which does not exist. I believe it should point to F2b red. Also the reference to fi seems wrong, fi denotes the node most to the right and about halfway up the positive axis. The red node does not seem to have a designation. How is that linked with the first subproblem?*

Response: Thank you for pointing out the typo. It should be **Fig. 2b** red.

Regarding the notation, here $f_i = f(P_i)$. f_i is the corresponding visual coordinate for point P_i. P_i is approximately at the center of the polygon in **Fig. 2a**. We’ve listed three cases for f_i: (1) if f_i is located in the center of the polygon in **Fig. 2b** and makes the triangle $\Delta f_i f_j f_k$ similar to $\Delta P_i P_j P_k$, then $|\mu| = 0$; (2) if f_i moves a little bit, that makes $|\mu| < 1$; (3) However, if f_i moves below the x-axis (like the red dot), then $|\mu| > 1$. In case 3, the orientation of triangle $\Delta P_i P_j P_k$ is different from that of $\Delta f_i f_j f_k$. The Beltrami coefficient provides a metric to quantify and monitor the orientation (if all the orientation are right the topological condition holds).

In our formulation, the first subproblem is smoothing, which smooths the visual coordinates, but cannot guarantee the topological condition. The second subproblem is topological projection, which ensures that the processed visual coordinates are topological. Both subproblems work on the visual coordinates. As discussed in response to **Q2.9**, they are directly connected. The Beltrami coefficients encodes the topological condition and can be used to reconstruct the mapping (theoretical background is given in SI 2). The topological projection updates those non-topological part (triangles with $|\mu| > 1$) to satisfy the topological condition. By solving these two subproblems iteratively, one may obtain both smooth and topological retinotopic maps.

Revision: We've modified **Fig. 2** to make it clearer and changed the subtitle.

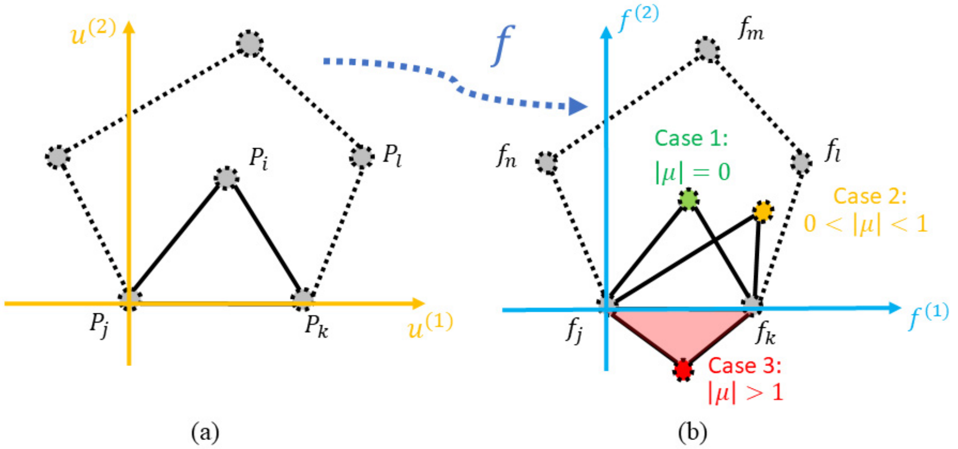

Figure 2. Beltrami coefficient and topological condition: **(a) source triangle (triangle $\Delta P_i P_j P_k$), (b) three target triangles $\Delta f_i f_j f_k$ from three different types of mappings, with different Beltrami coefficients.**

We've also made it clear about the subproblems:

"To solve Eq. (4) efficiently, we divide the problem into two subproblems, namely smoothing and topological projection. Specifically, during smoothing, we ignore the topological condition and find a smoothed map by,

$$\tilde{v} = \arg\min_{\tilde{v}} \int w|\tilde{v} - v|^2 + s|\nabla\tilde{v}|^2 ds \quad (6)$$

which can be solved by Laplacian smoothing [44,45] efficiently. For the second subproblem, we ignore the smoothness term and try to make the results topological.

$$\hat{v} = \arg\min_{\hat{v}} \int w|\hat{v} - \tilde{v}|^2 ds \text{, s.t.} \|\mu_{\hat{v}}\|_\infty < 1 \quad (7)$$

Since we require the results to be both topological and smooth, we solve the subproblems iteratively. Namely, the output of topological projection is fed to smoothing, and so on so forth."

Q2.12 *L560F "angle distortion". Angle distortion is typically called anisotropy, and yes, this has been "formally formalized". It is an extremely common topic in the field of vision science with many authors discussing and analysing it, even Holmes and Lister (1916) were discussing it. The pattern anisotropy at eccentricities 0.5 and 1 degree displayed in **Fig. 13** looks suspiciously like the one found by Schira et al. (2007, 2009 and 2010). The asymmetry for eccentricity ranges 1.5 and 2 degrees (yellow and purple curve) however has not been reported before. However, if the authors would like to present these results, they need to deliver a much more solid report on how it was obtained, they need to provide an analysis of the variance (i.e. error bars…) across subjects and make sure the results are not the consequence of a systematic error. Just glancing at these I would be very suspicious the anisotropy found at -pi/2 (ventral?) but not at pi/2 (dorsal?) could be a systematic error, as nothing like this has ever been reported. Extraordinary claims require extraordinary evidence.*

Response: It is a reasonable question. As discussed in response to **Q2.1**, we've deleted the angle distortion subsection in the revised manuscript. We hope to present our in-depth research on this topic in the future.

Here we want to take this opportunity to clarify the difference between anisotropy and angle distortion because we also used angle distortion in our synthetic simulations. First of all, anisotropy, to our knowledge, is about changes of the properties of the retinotopic map behavior along different directions. As suggested by the reviewer, the anisotropy of retinotopic maps was discovered many years ago. Although related, angle distortion in our discussion is different from anisotropy.

Let us assume that we have a retinotopic map that maps an infinitesimal circle in visual space to an infinitesimal ellipse on a 3D cortical surface. Since the ellipse is very small, we can assume that the 3D cortical surface is flat in this tiny region. We can cut this tiny cortical region out and place it on the disk (possibly with uniform scaling).

Anisotropy quantifies the eccentricity of the ellipse by taking the ratio of longest axis to the shortest axis, K. If $K = 1$, a circle is mapped as circle. Although the Beltrami is used to monitor topological condition in this paper, we shall emphasize that Beltrami can also be used to compute anisotropy: $K = \frac{1+|\mu|}{1-|\mu|}$ (see SI 2 for an example).

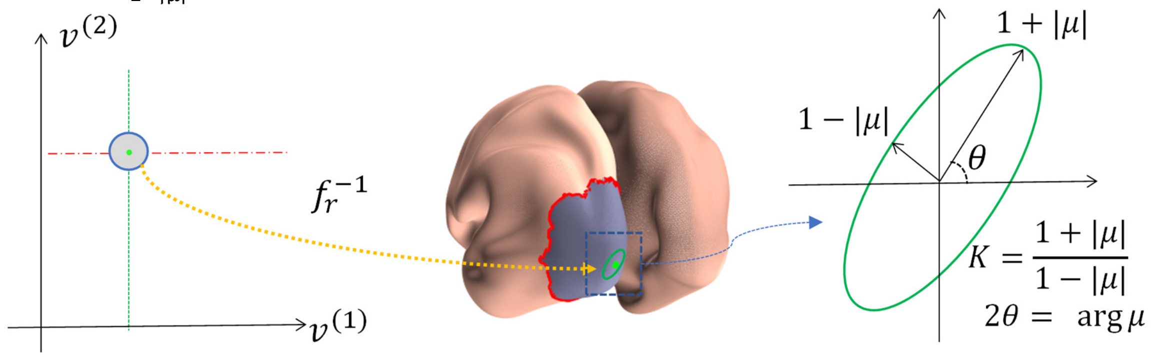

(a) (b) (c)

Angle distortion is a different concept. Assume that the tiny circle moves along the red horizonal dashed line, back to the original position and moves along the green vertical dashed line, the center of the ellipse will also move on the corteical surface and form two traces on the cortical surface (red and green curves in the figure below). Angle distortion is equal to $|\pi/2 - \alpha|$ ($\pi/2$ is the angle formed in visual space).

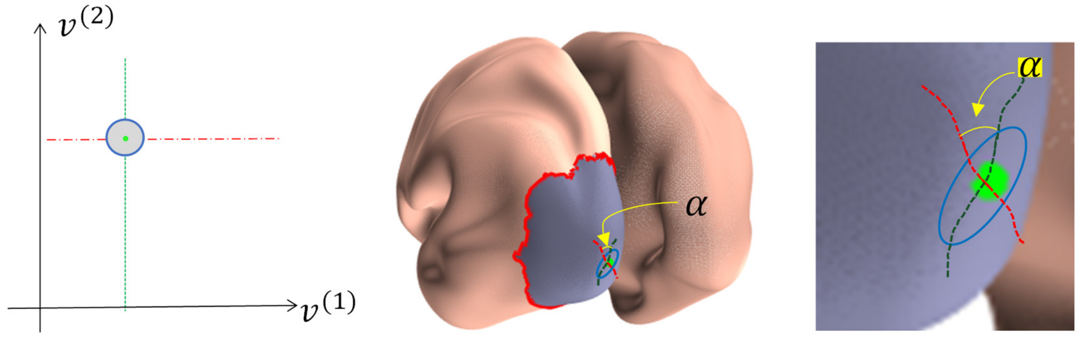

(a) (b) (c)

Angle distortion is related to the anisotropy. The relationship between angle distortion and anisotropy has been added in SI 4. When the retinotopic map is conformal, one can use anisotropy to directly infer angle distortion.

We further show the relationship between angle distortion $\delta_\angle$ and the Beltrami coefficient $\mu = \rho + i\tau$. Using the definition of μ, we can eliminate $\partial f^{(1)}/\partial u^{(2)}$ and $\partial f^{(2)}/\partial u^{(2)}$ with the terms of $\partial f^{(1)}/\partial u^{(1)}$ and $\partial f^{(2)}/\partial u^{(1)}$. Eventually, angle distortion can be written as:

$$\delta_\angle = \frac{\pi}{2} - \arccos\left(\frac{2\,(k^2\rho^2\tau + k^2\tau^3 - k^2\tau + 2k\rho^3 + 4k\rho^2 + 2k\rho\tau^2 + 2k\rho + 4k\tau^2 - \rho^2\tau - \tau^3 + \tau)}{(\rho^2 + 2\rho + \tau^2 + 1)^2 + (k\rho^2 + k\tau^2 + 2\tau - k)^2}\right),$$

where $k = \frac{\partial f^{(2)}/\partial u^{(1)}}{\partial f^{(1)}/\partial u^{(1)}}$.

In general, μ cannot be used to determine angle distortion $\delta_\angle$ without k. **However, the anisotropy ratio can be determinate by μ directly: $K = (1 + \rho^2 + \tau^2)/(1 - \rho^2 - \tau^2)$.** In particular, angle distortion is $\delta_\angle = 0$ and $K = 1$ when $\mu = 0$ (when the retinotopic map is conformal).

To our knowledge, there still lacks efficient and accurate methods to characterize local angle distortion. Surface conformal mapping provides a way to alleviate the discussion. Our conformal parametrization preserves angle after parametrization and allows us to discuss it on 2D.

We plan to study this and related issues further in our future work.

Revision: We added the relation between anisotropy and angle distortion in the SI 4, and removed the angle distortion section.

*"**Relation to anisotropy***

Angle distortion is closely related to anisotropy, but not identical. Let us assume that we have a retinotopic map that maps an infinitesimal circle in visual space to an infinitesimal ellipse on a 3D cortical surface. Since the ellipse is very small, we can assume that the 3D cortical surface is flat in this tiny region. We can cut this tiny cortical region out and place it on the disk (possibly with uniform scaling).

Anisotropy quantifies the eccentricity of the ellipse by taking the ratio of longest axis to the shortest axis, K. If $K = 1$, a circle is mapped as circle. Although the Beltrami is used to monitor topological condition in this paper, we shall emphasize that Beltrami can also be used to compute anisotropy: $K = \frac{1+|\mu|}{1-|\mu|}$ **(Fig. S3).**

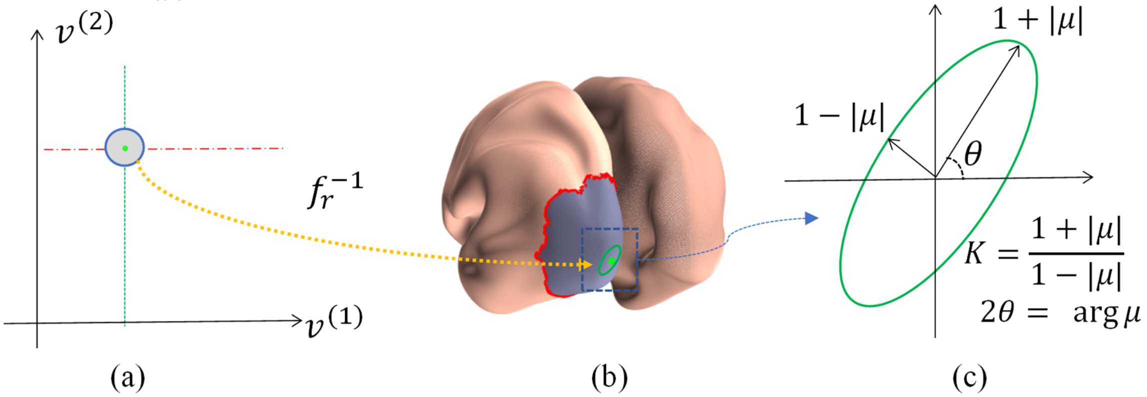

Figure S3. Illustration of anisotropy in retinotopic map and its relation to the Beltrami coefficient.

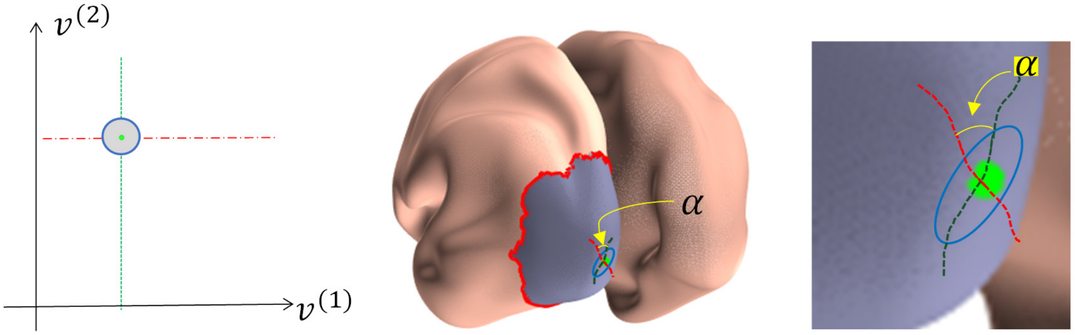

(a) (b) (c)

Figure S4. Illustration of angle distortion in retinotopic map.

Angle distortion is related to the anisotropy. Only if the retinotopic map is conformal, one can use anisotropy to directly infer angle distortion.

We further show the relationship between angle distortion $\delta_\angle$ and the Beltrami coefficient $\mu = \rho + i\tau$. Using the definition of μ, we can eliminate $\partial f^{(1)}/\partial u^{(2)}$ and $\partial f^{(2)}/\partial u^{(2)}$ with the terms of $\partial f^{(1)}/\partial u^{(1)}$ and $\partial f^{(2)}/\partial u^{(1)}$. Eventually, angle distortion can be written as: $\delta_\angle = \frac{\pi}{2} - \arccos\left(\frac{2\left(k^2\rho^2\tau + k^2\tau^3 - k^2\tau + 2k\rho^3 + 4k\rho^2 + 2k\rho\tau^2 + 2k\rho + 4k\tau^2 - \rho^2\tau - \tau^3 + \tau\right)}{(\rho^2 + 2\rho + \tau^2 + 1)^2 + (k\rho^2 + k\tau^2 + 2\tau - k)^2}\right)$, where $k = \frac{\partial f^{(2)}/\partial u^{(1)}}{\partial f^{(1)}/\partial u^{(1)}}$.

In general, μ cannot be used to determine angle distortion $\delta_\angle$ without k. **However, the anisotropy ratio can be determinate by μ directly: $K = (1 + \rho^2 + \tau^2)/(1 - \rho^2 - \tau^2)$**. In particular, angle distortion is $\delta_\angle = 0$ and $K = 1$ when $\mu = 0$ (when the retinotopic map is conformal)."

Q2.13 *L39 "The violation of topology condition is especially severe near the fovea, "this remains a little fuzzy, not specified what 'near the fovea' means, like 1 degree eccentricity?*

Response and Revision: From our experience, the pRF model has difficulty to accurately decode visual coordinates near the fovea, due to the limited spatial resolution of the fMRI signals and other issues. However, the exact size of the affected neighborhood depends on the data quality. In our current experiment with the HCP dataset, data in less than 1 degree eccentricity are typically noisier and contain more topological violations.

We've updated the sentence to make the specification clearer.

"**The violation of topological condition is most severe in cortical regions corresponding to the neighborhood of the fovea (e.g., < 1 degree eccentricity in the Human Connectome Project (HCP) dataset), …**"

Q2.14 *L62 "recent advances in Blood Oxygenation Level Dependent…" This statement feels a little bit out of time. These "recent" advances have allowed retinotopic mapping and estimation of magnification more than 25 years ago by several authors (Sereno et al, De Yoe et al, Engel et al.) "recent advances" is a phrase commonly used to highlight sub mm fMRI, and even in this field the phrase feels dated. This somewhat extends to the entire paragraph. In the last two decades many more visual areas than just V1, V2 and V3 have been discovered.*

Response and Revision: Thanks for the suggestion. We did the following modifications:

"Although initially discovered in neurophysiology [2], **Blood Oxygenation Level dependent (BOLD)** functional magnetic resonance imaging (fMRI) provides..."

"In the last two decades, **the development of a variety of retinotopic mapping techniques has greatly extended our** understanding of visual cortical organization in normal and abnormal populations [3,8–13]. "

Q2.15 *L120 suggests fi is "a mapping". I find this confusing and suspect it's wrong. I also do not understand how fi can be "moved out of any of the edges" (L121). The way it looks to me, in F1c the edge fjfk (or the triangle fjfkfi) violates the surface. The authors really need to clarify their terminology here.*

Response and Revision: We apologize for the confusion.

Here we use f_i to denote the mapping result of point P_i on the retina space, i.e., $f_i = f(P_i)$. We've changed in both Figure 1 caption and text by

"Namely, one may check whether its mapping **result**, f_i, is moved out of any of the polygon edges, $\overline{f_j f_k}$, $\overline{f_k f_l}$, $\overline{f_l f_m}$, $\overline{f_m f_n}$, and $\overline{f_n f_j}$. If f_i is moved out of any of the edges, **e.g.,** $\overline{\boldsymbol{f_j f_k}}$**,** the mapping is not topological.**"**

As we explained in Response to **Q2.2**, the topological condition discussed here is not related to genus or handles. In our case, we know the edges/faces are completely fine for the cortical mesh, and the violations in visual mapping are caused by visual coordinates. Therefore, we did not update the edges/faces, but fixed their visual coordinates.

Q2.16 *I suspect the authors may understand F1 as an illustration of a surface to surface mapping, where F1b) is the retina and F1c) is the cortical surface. Then for the node fi the topology of the mapping is violated. However, then F1a) is confusing and the entire Figure 1 does not consider the retina a part of the mapping. Then the triangle PiPjPk is in the retina/visual space and the tringle fifjfk in cortex?*

Response: In **Fig. 1**, (a) is on the cortical surface and both (b) and (c) are in the retinal domain. (a) is used to illustrate the original topological relationship of points, P_i, P_j, P_k, in the source (inflated cortical surface) domain. Retinotopic mapping assigns visual coordinates to each of them. The assigned visual coordinates are either topological (b) or non-topological (c). (c) is an example of non-topological mapping. Numerically, we can compute the Beltrami coefficient, μ, of the mapping $\Delta P_i P_j P_k \rightarrow \Delta f_i f_j f_k$. By checking the magnitude value of μ, one can learn whether the mapping is topological ((b), $|\mu| < 1$) and non-topological ((c), $|\mu| > 1$).

Revision: We have carefully updated **Fig. 1** caption to better explain the picture.

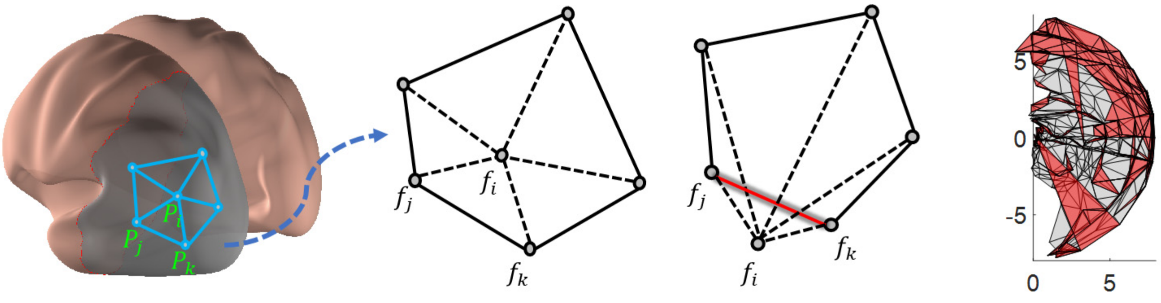

(a) (b) (c) (d)

Figure 1. Illustration of topological and non-topological **maps**. (a) A polygon patch on an inflated cortical surface (source domain). (b) A topological mapping of (a) **in the target domain (retina space)**. It preserves the structure of the source polygon. (c) A non-topological mapping of (a). It breaks the structure of the source polygon: f_i is moved out of the edge $\overline{f_j f_k}$, resulting in what is called a *flipped triangle* $\Delta f_i f_j f_k$. (d) A typical retinotopic map of V1. Flipped triangles are labeled with red color.

Q2.17 L194 "assuming that the voxel's respective is …" what is a respective? I am not certain it is required for this manuscript to describe the equations of the pRF fitting procedure. A simple 4-5 line section with quoting the relevant manuscript describing the specific implementation used should suffice.

Response and Revision: It was a typo. We've changed it to "where β is the activation level**, n is the exponent of the power function. We used the standard population receptive field model, i.e., $r(v'; v, \sigma) = \exp\left(-\frac{(x'-x)^2+(y'-y)^2}{2\sigma^2}\right)$, where $x = v^{(1)} \cos v^{(2)}$, $y = v^{(1)} \sin v^{(2)}$ (similarly for x', y').**"

Further, since we used the R^2 metric in performance evaluation, we need to define it in the context of pRF model fitting. As Reviewer #3 suggested, the description of pRF was too short. In the revised manuscript, we briefly described the pRF model to introduce necessary notations.

Accordingly, we started the "pRF decoder" subsection by

"We briefly describe the population receptive field model (pRF) [3,38] to introduce some necessary notations. For readers familiar with pRF model, the visual coordinates we used here are related to the x-y coordinates by $x = v^{(1)} \cos v^{(2)}$, $y = v^{(1)} \sin v^{(2)}$"

Q2.18 *L291 "we now explain how to recovery…" should be "recover" I think.*

Response and Revision: Thank you for the suggestion. We fixed the typo and double checked grammar throughout the entire manuscript.

Q2.19 *L 595 "Quality assessment". I am not certain what that paragraph is supposed to illustrate, other than apparently that for S1 the method changed the map only a little, while for S2 it changed a lot. What does that mean? What about the other 169 subjects? What it too much what is too little? Of course, quality control is an important step in any data processing/analysis and hence a worthwhile discussion topic. The authors should try and define some sort of criteria/logic. For example the authors could provide a quantitative measurement of the amount of smoothing and report how these values are distributed. This would allow to classify good vs bad datasets and*

provide a framework to judge this. One could argue this would go beyond the scope of the publication, but if included it must be of substance.

Response: Thank you for the suggestion. The quality assessment basically quantifies the difference between the smoothed and raw data at each vertex. In **Fig. 13** of the original submission, we showed the distortion distribution before and after the smoothing, with the unit of visual angle, for the first two subjects. In the revised manuscript, we've added the average distortion distribution of all 181 subjects in **Fig. 12d**. We've also updated the results for all 181 observers in **Table 5**. We've updated the figures with results from all subjects in other sections.

Revision: We've revised the quality assessment subsection as below.

"*Quality assessment*

In this study, we enforced the topological condition in retinotopic mapping. This enforcement could potentially introduce large visual coordinate changes to the raw data compared to other smoothing methods. It is important to monitor whether the smoothing results are acceptable or have relatively big deviations. Such quality assessment can provide the right level of confidence when drawing conclusions based on the smoothing results. In Fig. 12, the visual coordinate changes of two typical observers are shown on the cortical and parametric surfaces respectively. Fig. 12a shows the studied visual areas on the 3D cortical surface. Fig. 12bc shows the distortion in visual space. The colors indicate the amount of visual coordinate changes for the proposed smoothing process. The first observer's results (Figs. 12a) suggested that the smoothing was generally good, with on average an about $1.12°$ change of visual coordinates. In contrast, the other observer's results (Figs. 12c) suggested that some sub-regions have relatively big visual coordinate changes, with on average an about $1.75°$ change of visual coordinates. We also show the mean visual coordination distortion distribution of all 181 subjects in Fig. 12d. Together with Table 5, the quality assessment results ensure our retinotopic mappings well respects the original fMRI signals.

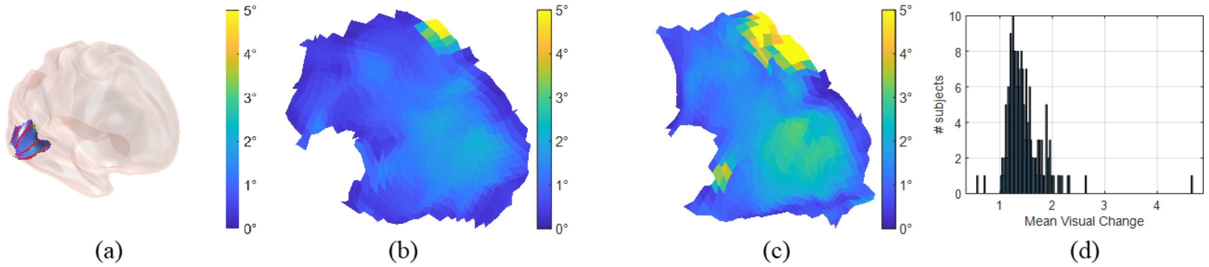

Figure 12. Visual coordinate change after smoothing. (a) The visual change is rendered on the inflated cortical surface (b) The visual change is rendered on the parametric surface for the first observer. Similarly, (c) is the visual change for the second observer. (d) is the histogram of average visual change for the HCP dataset (N=181)."

Table 5 (with all 181 observer data) has been updated.

Method	$\hat{\boldsymbol{T}}$	$\boldsymbol{Ar_n}$(%)	*NRMSE* $\times \mathbf{10^{-3}}$	$\overline{\boldsymbol{R^2}}$
Before Smoothing	73	20.7	**0.07757**	**28.23**
Average Smoothing	28	8.1	0.07779	27.86
Median Smoothing	35	9.7	0.07773	27.92
Laplacian Smoothing	64	18.0	0.07763	28.09
Proposed Smoothing	**0**	**0.0**	0.08139	20.35

Table 5. Comparison of different methods in fitting the fMRI time series.

We added one discussion subsection for **Table 5**:

"***Elaboration on the reduced R^2 in Table 5***

The topological smoother can completely remove topology violations in retinotopic maps. However, as shown in Table 4, it reduced the measure of explained variance, R^2. In general, the pRF model should have a better R^2, since R^2 values are computed at each point on the cortical surface. There are no constraints, nor penalty for non-topological results in R^2. However, neurophysiology studies strongly suggested that retinotopic mapping is topological for normal subjects. If the pRF results are not topological, the problem is very likely caused by the low spatial resolution and SNR of the fMRI data, an issue that cannot be addressed with current MRI technologies. In this work, we assume that the neurophysiology results are correct and attempted to fix topological violations in retinotopic maps by slightly updating the visual coordinates. We used pRF results as input data without 1) fine tuning pRF model parameters, such as the gain or the exponent of the power function [61], and 2) iterating with the pRF model to search for an optimal solution with balanced fitting quality and topological condition. Our ongoing work [61] tries to integrate the pRF model and topological conditions by defining a combined energy term. We expect such a balanced approach may improve topological condition without sacrificing data fitting quality."

Reviewer #3: *This is a method manuscript. It tackles a very specific problem in population receptive field (pRF) fitting algorithms. Basically, most of the pRF algorithms work at the voxel level, and per every voxel, the algorithm will provide the pRF center and size. By definition, those pRF centers need to be unique and retinotopically organized. Usually, these voxels are mapped to the surface, in a 2D surface mesh, so that every vertex corresponds to a time series. As these fMRI time series are very noisy, it can happen (in 20% of the cases according to the authors) that the pRF center values estimated from those time series break the topological condition. The authors propose a method to solve this issue, generating topology-preserving retinotopic maps. pRF algorithms are very powerful but they are relatively modern (they were introduced in 2008). There are improvement opportunities, and it is important for the field that the authors identify a weakness of the method and propose a solution to it. The paper is clearly written, it is well organized and the figures are generally clear, which is a great pleasure as a reviewer.*

Thanks for the encouragement.

Q3.1 *The authors published this method already in ISBI 2020 (https://www.ncbi.nlm.nih.gov/pmc/articles/PMC7406191/#!po=43.3333). I think that: (1) If the method has been significantly improved, the ISBI paper should be cited and here explain what those improvements consist of. (2) If there are no improvements on the methods but there are in the applications (CMF and angle distortion quantifications?, Discussion, examples...), the ISBI paper should be cited for the methods part and add the new contribution here. I can see how much work went into this manuscript, but it is the job of the editors to decide if the contribution is big enough to be published in their journal, but as a reader I would expect the other paper to be acknowledged/explained in this one.*

Response: Thanks for the question. It was a mistake for us not to cite our ISBI 2020 paper. We've cited it in the revised manuscript. Compared with the preliminary results reported in our ISBI 2020 paper, we've made the following improvements in the current work. *First,* our ISBI 2020 work was only applicable to one visual region, namely V1. More precisely, the V1 region inferred from structural registration. In the current study, we extended the work to the V1-V3 complex. Relying on the out-most visual coordinates, the extension made it possible to delineate V1/V2 and V2/V3 boundaries. It also generated more precise smoothing results within the interior of each visual area. Therefore, we used a more general name, "topological smoothing", to describe our work since the mapping from several visual regions to the retina is no longer diffeomorphic, but topology preserving in the extended work. *Secondly*, our ISBI 2020 work was mainly evaluated by the changes of the visual coordinates, but how much impact it had on the goodness of fit of the fMRI time series was not evaluated. Here we added fits to the fMRI time series in performance evaluation, which may better justify our work and alleviate the concern that the proposed method may over-smooth the retinotopic maps. Thirdly, we've also added more experimental details and discussions, e.g., boundary conditions and parameter selection, etc. Those details are impotant to reproduce our results. Finally, we also applied the algorithm to all the HCP retinotopic map data (N=181) by improving the accuracy and stability of the algorithm, especially the interior boundary conditions.

Revision: We've properly cited and acknowledged the ISBI work in Introduction section, and explained the main differences.

"The topological condition was considered in our previous work [29], but it was only applied to V1 with limited validation. And, because the manually drawn boundaries were not accurate, the results were not very precise near the boundary."

We also added a discussion subsection to explain the main differences between ISBI 2020 and our current work.

"Benefits of the extended polar angles
Our previous work on topology correction [29] was only applicable to V1; it could not be used to improve boundary delineation. In this work, by introducing extended polar angles, we extended the framework to the V1/V2/V3 complex and achieved topology-preserving smoothing in multiple visual areas simultaneously. Although our previous work [29] might be potentially appliable to V2 and V3, accurate V1/V2 and V2/V3 boundaries were necessary. In the current work, given the out-most boundaries of the V1/V2/V3 complex, our work not only generated more precise smoothing within the interior of each region, but also automatically delineated the boundaries between them. The new development on boundary delineation may significantly improve the robustness and accuracy of retinotopic mapping. In addition, our prior work was mainly evaluated with visual coordinate the changes. How much impact it had on the goodness of fit of the fMRI time series was not evaluated. Here we added fits to the fMRI time series in performance evaluation, which may better justify our work and alleviate the concern that the proposed method may over-smooth the retinotopic maps. With extensive evaluation based on both synthetic data and real retinotopic map data from 181 HCP observers, the current study demonstrated that our proposed method may improve the accuracy and stability of retinotopic mapping, especially the interior boundary conditions."

Q3.2 *Synthetic data: If I understand it correctly, you basically take a topology and map an invented set of pRF center parameters, with more or less noise. I don't understand why is the topology cuadricular when the vertices in the cortex are organized in triangles. I guess they are "equipotential" (angle, eccen) lines and not the triangles of the surface?*

Response: Your understanding is correct. They are equipotential curves (contours of angle or eccentricity). For the synthetic data, we created triangles from the grid of equipotential curves for processing convenience.

Revision: We made the following clarification in the synthetic data subsection:

"More specifically, the visual field grid mesh is first created (Fig. 7a) with the following configurations: eccentricity spans from $0°$ to $4.5°$ and polar angle ranges from $-\pi/2$ to $\pi/2$. We then sampled 12 points in each direction and formed the quadrilateral grid mesh (144 points in total). Based on the quadrilateral mesh, we created the triangular faces by connecting diagonal points for each quadrilateral. Then we mapped the triangular mesh to the parametric space (Fig. 7b), according to Eq. (13). Ideally, the mapping points should be located on the grid (Fig. 7b). We then added a small amount of noise with a peak signal-noise ratio (PSNR) of 10 (Fig. 7c), and a large amount of noise with a PSNR $= 5$ (Fig. 7d) ."

Q3.3 *How can you prove that those modifications are realistically? I think that it would make more sense simulating time series with known ground-truth values (using Lerma-Usabiaga 2020 Plos Comp. Biol., for example) and adding noise until 20% of the triangles are flipped. And then applying the correction methods. You would know the ground truth of the parameters to estimate and therefore quantify the correction of your method.*

Response: Thank you for this excellent suggestion. In our original synthetic data experiment, we focused on perception centers and added white noise to them. Such a setting had its benefits -- it was straightforward and did not involve specific pRF decoding model selection and its settings. However, as suggested by the reviewer, a potential question is whether the white noises we added were realistic.

To evaluate the performance of our proposed method in realistic fMRI noises, we've added a new set of experiments following the reviewer's suggestion. The procedure is as follows.

1. We started with the ground-truth parameters: perception centers spanning the right visual space $[0:0.08:6.4]° \times [0: 0.08: 4.8]°$; receptive field size = 0.4°; gain = 1; and power of exponent $n = 0.5$.
2. Followed Kay's pRF model (visual stimulus movie from the HCP and spatial resolution of decoding stimulus is 200×200 with 1800 seconds) to generate the ideal fMRI signal.
3. Normalized the fMRI signal to unit variance. Then added a specific amount of white noise. Decoded the pRF model for the fMRI signal (with noise) and computed the number of flipped triangles.
   a) Repeated step 3 with different noise levels until the added noise made the percentage of flipped triangle to be about 20%. (Specifically, in our experiments, noise with a 2.5 standard deviation induced ~22% flipped triangles). Note that the flipped triangles were identified after decoding the pRF model. Therefore, it is not feasible to precisely control the number of flipped triangles before decoding. Besides, the PSNR for the visual coordinate change was 6dB, within the range of 5~10dB in our previous discussion.
   b) With such pRF decoding results as input, we applied the topological smoothing method and generated new visual coordinates. Then we compared our work with the pRF model and several post-processing methods **(Table 4)**. We found that (1) the proposed method can fully ensure the topological condition, with **0** flipped triangles, while other smoothing methods had at least 6% of flipped triangles not being fixed; (2) the proposed method had minimal angle distortion; and (3) all smoothing methods improved the perception center estimates, with the median smoothing method achieving minimal distortion. The last point is reasonable because the current work is mainly focused on the topological condition. Future fine-tuning of the pRF parameters may alleviate this problem.

The new synthetic experimental results further demonstrated that the proposed method could remove topology violations and reduce angle distortions.

Revision: We added a new experiment as the second synthetic experiment in the revised manuscript.

"***Synthetic Data II***

We performed another set of synthetic data experiment in which noise was directly added to the fMRI signals. We started with the ground-truth parameters: receptive field centers spanning the right visual space [0:0.08:6.4]° × $[\mathbf{0}: \mathbf{0.08}: \mathbf{4.8}]$°; receptive field size = 0.4°;

gain = 1; and exponent of the power function $n = 0.5$. Then, we followed Kay's pRF model (AnalyzePRF [38,55] with stimulus from the HCP group, 200×200 in spatial resolution with 1800 seconds) to generate the ideal fMRI signal. We further normalized the fMRI signal to unit variance, added a specific amount of white noise, decoded the pRF model for the fMRI signal (with noise), and computed the number of flipped triangles. We varied the noise level until the percentage of flipped triangle was about 20%. (Specifically, in our experiments, noise with a 2.5 standard deviation induced about 22% flipped triangles). With pRF decoding results at this noise level as input, we applied the topological smoothing method and generated new visual coordinates. Then we compared our work with the pRF model and several post-processing methods (Table 4).

**Method**	**Center Distortion**			**Angle Distortion (degree)**			$\hat{T}$	R^2
	**Mean**	**Std**	**p**	**Mean**	**Std**	**p**		
**No Smoothing**	**0.407**	**0.520**	**0.352**	**48.55**	**30.20**	**0.00**	**32**	**13.28**
**Average**	**0.311**	**0.238**	**0.070**	**23.35**	**23.16**	**0.00**	**15**	**13.41**
**Median**	**0.298**	**0.176**	**0.010**	**22.40**	**16.57**	**0.00**	**11**	**13.41**
**Laplacian**	**0.429**	**0.509**	**0.149**	**42.82**	**29.48**	**0.00**	**23**	**13.09**
**Proposed**	**0.361**	**0.231**	**------**	**13.84**	**12.87**	**------**	**0**	**13.27**

Table 4. Comparison of different smoothing methods based on noisy fMRI decoding with about 22% of flipping triangles.

We found that (1) the proposed method can fully ensure the topological condition, with 0 flipped triangles, while other smoothing methods had at least 6% of flipped triangles; (2) the proposed method had minimal distortion; and (3) all smoothing methods improved the receptive field center estimates, with the median smoothing method achieving minimal distortion. The last point is reasonable because the current work is mainly focused on the topological condition. Future fine-tuning of the pRF parameters may alleviate this problem. The new synthetic experimental results further demonstrated that the proposed method could remove topological violations and reduce angle distortions."

Besides, the synthetic figure is updated with results with PSNR =5:

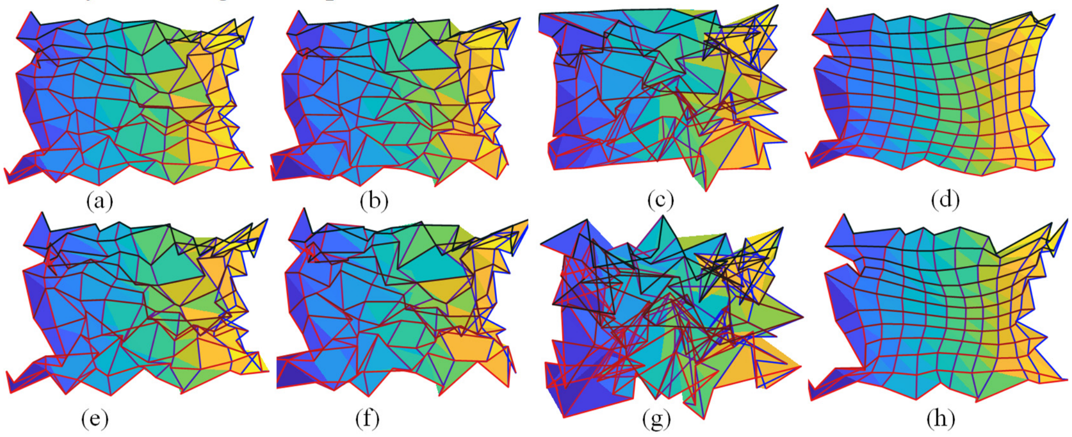

Figure 8. Smoothing results for the synthetic data: (a)-(d) are the results with PSNR=10 for (a) Average smoothing, (b) Median smoothing, (c) Laplacian smoothing, and (d) proposed topological smoothing; **(e)-(h) are the results with the same order as (a)-(d) but with PSNR=5.**

Q3.4 *How can you know that typically 20% in an experimental dataset are flipped?* ***Table S2****?*

Response: Yes, one can check the total number of triangles in V1 and the flipped triangles in **Table S2** for the first five subjects. For more subjects, one can check the OSF repository. In general, there are more than 20% of flipping triangles in the whole HCP dataset.

Revision: We've added a reference to the relevant table: "with about 20% of the triangles flipped **(see Table S2),** is illustrated in **Fig.** 1d."

**Observers**	**Raw**	**Ave.**	**Med.**	**Lap.**	**Ours**
**S1**	118	55	63	63	**0**
**S2**	124	66	77	77	**0**
**S3**	90	24	34	34	**0**
**S4**	93	7	16	16	**0**
**S5**	102	18	43	43	**0**

Table S2. The number of flipped triangles for the first five observers after smoothing with different methods. The total number of triangles is 548.

Q3.5 *Experimental data: I don't think the R2 calculations are the adequate ones here… Are you basically saying that for the time series, the solver went to a local minima but that there was a better solution, and that your algorithm could find the better fit to the time series? Knowing how most of the algorithms work (seed > grid search > fine search), this can happen, but maybe there are better ways to show this than the one you provide. And if they are not, the concept you think how it is going on should be explained, I think.*

Response: It is a reasonable concern. During the revision, we processed the retinotopy data from all the 181 HCP observers. We also fixed some processing errors, so we have updated both NRMSE and $\overline{R^2}$ (the average of R^2) results in **Table 5** (the original **Table 4**). In addition, we now report R^2 for the newly added synthetic data experiment in **Table 4**. We found that our proposed method achieved a similar R^2 in the synthetic experiment (**Table 4**) and a reduced R^2 in the HCP data experiment compared to the other methods (**Table 5**).

We did not re-fit the pRF model in this study. We only used the new visual coordinates but kept all the other pRF parameters (e.g., gain, and exponent power) unchanged when we computed the fMRI time series predictions.

Revision: For the newly added experimental results (**Table 4**), we noted that the R^2values are declined.

For V1 retinotopic map results (**Table 5**), We emphasized the parameter setting details and the purpose of our evaluation.

"To evaluate the impact of changing the receptive field centers, we set the response level g and the exponent of the power function n the same as the original pRF in computing fMRI time series predictions.

We measured the goodness of fit of the smoothing methods to the fMRI time series (with the code from AnalyzePRF [38,55])."

In the results part, we've added

"(3) The proposed method decreased R^2, mainly due to the constrain of the topological condition. The number of flipped triangles for individual observers by all methods can be found in the supplementary data (see *SI 6*). The results also show that the

proposed method was always better in preserving topology while the goodness of fit to the fMRI time series was reduced from the original result."

In Discussion section, we added a subsection, "***Elaboration on the reduced R^2 in Table 5"*** (to be detailed in the answer to **Q3.6**).

"**In this work, we assume that the neurophysiology results are correct and attempted to fix topological violations in retinotopic maps by slightly updating the visual coordinates. We used pRF results as input data without 1) fine tuning pRF model parameters, such as the gain or the exponent of the power function [61], and 2) iterating with the pRF model to search for an optimal solution with balanced fitting quality and topological condition. Our ongoing work [61] tries to integrate the pRF model and topological conditions by defining a combined energy term. We expect such a balanced approach may improve topological condition without sacrificing data fitting quality."**

Q3.6 ------ *How can we know that the fixed data is closer to the truth? We are modifying the estimated parameters according to one criterion: we don't want to have more flipped triangles. Do we need to assume that the averaged improved R2s are saying that we are closer to the truth or we are only saying that now we don't have flipped triangles only?*

Response: Thank you for the insightful question. In general, the pRF model should have a better R^2, since R^2 values are computed at each point. There are no constraints, nor penalty for non-topological results in R^2. However, neurophysiology studies strongly suggested that retinotopic mapping is topological for normal subjects. If the pRF results are not topological, the problem is very likely caused by the low spatial resolution and SNR of the fMRI data, an issue that can not be addressed with current MRI technologies. In this work, we assume that the neurophysiological results are correct and attempted to fix the topological violations in retinotopic maps by updating the visual coordinates.

Revision: We added one discussion subsection:

"***Elaboration on the reduced R^2 in Table 5***

The topological smoother can completely remove topology violations in retinotopic maps. However, as shown in Table 4, it reduced the measure of explained variance, R^2. In general, the pRF model should have a better R^2, since R^2 values are computed at each point on the cortical surface. There are no constraints, nor penalty for non-topological results in R^2. However, neurophysiology studies strongly suggested that retinotopic mapping is topological for normal subjects. If the pRF results are not topological, the problem is very likely caused by the low spatial resolution and SNR of the fMRI data, an issue that cannot be addressed with current MRI technologies. In this work, we assume that the neurophysiology results are correct and attempted to fix topological violations in retinotopic maps by slightly updating the visual coordinates. We used pRF results as input data without 1) fine tuning pRF model parameters, such as the gain or the exponent of the power function [61], and 2) iterating with the pRF model to search for an optimal solution with balanced fitting quality and topological condition. Our ongoing work [61] tries to integrate the pRF model and topological conditions by defining a combined energy term. We expect such a balanced approach may improve topological condition without sacrificing data fitting quality."

Q3.7 *Boundary definition: do we need to set the boundaries manually to initialize the method or this is only necessary for the tests in the paper? I think it would be very useful for the reader/final user that you would add a small "user guide". I understand that the user continues with exactly the same pipeline he/she was using, and then this method is something that is applied on top of it. After reading the manuscript, the manual and automated, the old and the new steps are not perfectly clear.*

Response: Thanks for the great question. There are two different scenarios.

1. The interior V1/V2 and V2/V3 boundaries were initially inferred from registration of subjects' cortical surface to the retinotopy atlas using Multimodal Surface Matching. These initial boundaries may not be accurate, but our algorithm can fix them by updating interior visual area labels.
2. The out-most visual boundaries of the V1/V2/V3 complex were pre-created on the fs_LR (the HCP used this space) sphere. One can use the pre-defined cortical boundaries and their corresponding visual coordinates as the out-most visual boundaries. We also provided a simple utility to create cortical boundaries and their corresponding visual coordinates, if one wishes to draw new boundaries. We've provided a short user manual on the OSF website.

Essentially, our work is applicable to new data starting with Freesurfer's or Multimodal Surface Matching registration outputs. All the data in this work had already been aligned in the fs_LR space.

Revision: We updated the "Boundary condition" subsection in Discussion.

"Out-most Boundary condition

In this work, visual areas, V1/V2/V3, and the initial boundaries between them were determined by surface registration. Following the HCP protocol [36], MSM [57] was adopted to register each observer's cortical surface to the retinotopy atlas obtained from the HCP [34]. After the registration, the boundaries for each observer were determined along equal polar angle curves, and our extended polar angles were initialized with these boundaries. The out-most eccentricity boundaries were manually determined based on the eccentricity signal range (in our case, approximately from $0.2°$ to $7°$).

The initial boundaries might not be accurate since they were determined by surface registration. With extended polar angles, our algorithm was robust enough to fix the interior visual area boundaries. Our method requires that the visual coordinates of the out-most boundaries are specified when solving the LBS. Even so, if the visual coordinates of the out-most boundary are not properly given, we can still update the out-most boundaries and recover interior visual coordinates. We tested two approaches to set the out-most boundary values. One approach is to set a fixed boundary for all observers to simulate the case in which a good boundary configuration is given, and the other approach is to adjust the out-most boundary values by fitting a smooth spline of the interior visual values to simulate the case in which the boundary coordinates are given but not precise. Each approach has its own pros and cons. The first approach is numerically stable but ignores individual differences, while the second approach adjusts the boundary values based on retinotopic data, which has higher accuracy but may fail to set reasonable boundary values. We compared both approaches in the experiments. Figs. 13ac are the results from the first approach, and Figs. 13bd are the results from the second approach. We found a relatively large difference on the

out-most boundaries (red boundaries in Figs. 13ab), while the interior regions were less affected. The mean difference between the results of the two approaches was $0.23° \pm 0.15°$, which is relatively small. In this paper, we took a combined approach: the boundary values were set within a small tolerance range ($0.5°$) of the initial average boundary values. Nevertheless, we found that, although the out-most boundaries may not be precise, the interior results were not much impacted by the boundary values. Therefore, we can use the pre-defined boundary configuration for all the subjects.

It is worth noting that our method is applicable to the registration results from Freesurfer [37] or MSM [57] or Bayesian register [8]. Here we applied it to successfully process data from all 181 HCP observers. In our code repository, we have published all the necessary manuals and atlas for others to reproduce our work. We also provided a simple software tool to manually draw the out-most boundaries on visual cortical surfaces and generate corresponding visual coordinates."

Q3.8 *pRF Methods are not explained sufficiently: it seems from the text, as Dumoulin and Wandell 2008 is cited (Page 8, Line 168: Page 9, Line 192), that the authors use mrVista software to fit the time series, but in a second lecture, it seems that they use the same algorithm used in the HCP 7T publication, which I guess is Kendrick Kay's AnalyzePRF?. I think that the software used should be explained specifically and maybe linking to publications/software repositories and versions, for clarity and reproducibility.*

Response and Revision: Thanks for the suggestion. Yes, we used Kay's AnalyzePRF model. In the revised manuscript, we've fixed the citation by adding Kay's compressive model. For reproducibility purpose, we used the initial pRF results from (https://osf.io/bw9ec/wiki/home/) (citation [20] has this link), where they used Kay's AnalyzePRF V1.1 to decode the pRF results. In the model fitting, we also used the Kay's AnalyzePRF V1.1 to evaluate the goodness of fit.

The following modifications have been made:

1. We've added a citation to Kay when we introduce the pRF model.
2. We've double checked the expression in **Eq. 1**, and added the exponent power *n* for Kay's model.
3. We've added references to Kay's code and version number in V2 retinotopic map subsection of Results.

   "We measured the goodness of fit of the smoothing methods to the fMRI time series (with the code from Kendrick Kay's AnalyzePRF [38,55])."
4. Reviewer #2 suggested that we remove the pRF descriptions. To balance the two different requests, we acknowledged the dataset and briefly explained the pRF model to introduce necessary notations.

Q3.*9 There are no papers cited later than 2018, but there have been relevant publications in the field not acknowledged by the authors (for example Lage-Castellanos 2020, or the previously cited Lerma-Usabiaga 2020). In the same line, I missed discussing other approaches to pRF fitting, such as the paradigm free approaches (Merkel 2018, 2020).*

Response and Revision: Thanks for this excellent suggestion. We've added more recent relevant works in Introduction.

“Typically, fMRI signals are analyzed on a voxel-by-voxel basis. The most prominent method is based on the population receptive field (pRF) model, which decodes perception parameters of the fMRI signals at each voxel. However, influenced by low SNR and low spatial resolution, the retinotopic maps from the analysis are typically not topological. In the past decade, significant efforts have been devoted to improving their accuracy and stability. For instance, the reliability of perception size was discussed [12,13], and methods that could decode perception parameters by model-free back projections with potentially more precise perception shape were proposed [21–23]. Although they have greatly improved the pRF model, none of the methods has explicitly investigated the topological condition.”

12. Lerma-Usabiaga G, Benson N, Winawer J, Wandell BA. A validation framework for neuroimaging software: The case of population receptive fields. Jbabdi S, editor. PLOS Comput Biol. 2020;16: e1007924. doi:10.1371/journal.pcbi.1007924
13. Lage-Castellanos A, Valente G, Senden M, De Martino F. Investigating the Reliability of Population Receptive Field Size Estimates Using fMRI. Front Neurosci. 2020;14: 825. doi:10.3389/fnins.2020.00825
21. Merkel C, Hopf JM, Schoenfeld MA. Modulating the global orientation bias of the visual system changes population receptive field elongations. Hum Brain Mapp. 2020;41: 1765–1774. doi:10.1002/hbm.24909
22. Merkel C, Hopf JM, Schoenfeld MA. Spatial elongation of population receptive field profiles revealed by model-free fMRI back-projection. Hum Brain Mapp. 2018;39: 2472–2481. doi:10.1002/hbm.24015
23. Lerma-Usabiaga G, Winawer J, Wandell BA. Population receptive field shapes in early visual cortex are nearly circular. J Neurosci. 2021; JN-RM-3052-20. doi:10.1523/jneurosci.3052-20.2021

Q3.10 *Page 2, Lines 44-45: that sentence is not correctly constructed.*

Response and Revision: Thanks, we updated the sentence to:
“The method was then applied to V1, V2, and V3 simultaneously in the HCP dataset.”

Q3.11 Ref 21 has an error it seems.

Response and Revision: Thanks. We’ve fixed it accordingly and checked other references.
